# A phase 2 study of an oral mTORC1/mTORC2 kinase inhibitor (CC-223) for non-pancreatic neuroendocrine tumors with or without carcinoid symptoms

**DOI:** 10.1371/journal.pone.0221994

**Published:** 2019-09-17

**Authors:** Edward Wolin, Alain Mita, Amit Mahipal, Tim Meyer, Johanna Bendell, John Nemunaitis, Pam N. Munster, Luis Paz-Ares, Ellen H. Filvaroff, Shaoyi Li, Kristen Hege, Hans de Haan, Monica Mita

**Affiliations:** 1 Experimental Therapeutics Program, Samuel Oschin Comprehensive Cancer Institute, Cedars-Sinai Medical Center, Los Angeles, CA, United States of America; 2 Phase 1 Clinical Trials Program, Mayo Clinic, Rochester, MN, United States of America; 3 Experimental Cancer Medicine, University College Hospital, London, United Kingdom; 4 GI Oncology Research, Sarah Cannon Research Institute/Tennessee Oncology, Nashville, TN, United States of America; 5 Department of Oncology, Mary Crowley Cancer Research Center, Dallas, TX, United States of America; 6 Early Phase Clinical Research Program, UCSF Helen Diller Family Comprehensive Cancer Center, San Francisco, CA, United States of America; 7 Medical Oncology Department, Hospital Universitario 12 de Octubre, CNIO, Universidad Complutense and CiberOnc, Madrid, Spain; 8 Translational Medicine, Celgene Corporation, San Francisco, CA, United States of America; 9 Department of Statistics, Celgene Corporation, Summit, NJ, United States of America; University of Wisconsin - Madison, UNITED STATES

## Abstract

Second-generation mammalian target of rapamycin (mTOR) inhibitors such as CC-223 may have theoretical advantages over first-generation drugs by inhibiting TOR kinase in mTOR complex 1 (mTORC1) and 2 (mTORC2), potentially improving clinical efficacy for well-differentiated neuroendocrine tumors (NET).Enrolled patients had metastatic, well-differentiated NET of non-pancreatic gastrointestinal or unknown origin, with/without carcinoid symptoms, had failed ≥1 systemic chemotherapy, and were taking a somatostatin analog (SSA). Oral once-daily CC-223 was administered in 28-day cycles starting at 45 mg (n = 24), with a subsequent cohort starting at 30 mg (n = 23). Objectives were to evaluate tolerability, preliminary efficacy, and pharmacokinetic and biomarker profiles of CC-223. Forty-seven patients completed the study, with mean treatment duration of 378 days and mean dose of 26 mg; 26% of patients remained on the starting dose. Most frequent grade ≥3 toxicities were diarrhea (38%), fatigue (21%), and stomatitis (11%). By investigator, 3 of 41 evaluable patients (7%) showed partial response (PR) and 34 (83%) had stable disease (SD) for a disease control rate (DCR) of 90% (95% confidence interval [CI] 76.9–97.3%). Duration of PR was 125–401 days; median SD duration was 297 days (min–max, 50–1519 days). Median progression-free survival was 19.5 months (95% CI 10.4–28.5 months). Carcinoid symptoms of flushing, diarrhea, or both improved in 50%, 41%, and 39% of affected patients, respectively. For the first time, this study describes that a second-generation mTOR pathway inhibitor can result in highly durable tumor regression and control of NET carcinoid symptoms. The manageable safety profile, high DCR, and durable response, coupled with reduction in carcinoid symptoms refractory to SSA, make CC-223 a promising agent for further development.

## Introduction

Neuroendocrine tumors (NET), although generally believed to be uncommon, are the second most common gastrointestinal (GI) malignancy, superseded only by colorectal neoplasias [1, 2]. This malignancy is reported to be increasing in prevalence, with the greatest increase in lung, small intestine, and rectal NETs [1]. Although survival has improved with introduction of newer therapies, prognosis for patients with advanced disease remains poor [1].

The phosphatidylinositol-4,5-bisphosphate 3-kinase (PI3K)–protein kinase B (AKT)–mammalian target of rapamycin (mTOR) signalling pathway, which is inappropriately activated in many cancers, plays a central role in the genesis and progression of NET. The mTOR complex 1 (mTORC1) inhibitor everolimus has become well-established as an effective therapy for NET arising from the pancreas, lung, or GI tract, with an associated median progression-free survival (PFS) of 11.0 months [3, 4]. mTOR kinase is a serine/threonine kinase that serves as a core component of two complexes; mTORC1 and mTORC2. The former modulates cell proliferation via phosphorylation of eukaryotic translation initiation factor 4E binding protein 1 (4E-BP1) and p70 ribosomal S6 kinase 1 (S6K1), whereas the latter acts via phosphorylation of AKT [5]. Everolimus inhibits the mTORC1 complex but may upregulate mTORC2, which thus maintains AKT activation. Consequently, inhibitors that can target both complexes may offer clinical advantages in terms of improved efficacy [5–10].

CC-223 is an inhibitor of TOR kinase in mTORC1 and mTORC2 that is known to overcome mTORC2 feedback pathway upregulation and thus potentially minimize development of therapy resistance due to increased phosphorylation of AKT [11]. In non-clinical studies, inhibition of mTORC1 and mTORC2 leads to more effective inhibition of cancer cell proliferation than does blocking mTORC1 alone [11]. In mice bearing human carcinoid xenografts that secrete serotonin, tumors that progressed on rapamycin remained stable or decreased in volume from baseline after treatment with CC-223 [12].

In a first-in-human study, CC-223 was evaluated as a treatment for patients with advanced solid tumors, and the 45 mg/d dose was established as the maximum tolerated dose (MTD) [13]. Here, we report results of an expansion cohort in patients with non-pancreatic GI or unknown primary NET treated at the previously-established MTD or lower dose if needed.

## Materials and methods

### Study design and participants

This open-label phase 1/2 study comprised two parts: dose-finding to determine maximum tolerated dose (phase 1) in a variety of solid and hematologic malignancies, the results from which have been previously published [13], and cohort expansion (phase 2). In phase 2, adults (≥18 years) with locally unresectable or metastatic well-differentiated (grade 1 or 2) non-pancreatic GI NET or NET of unknown primary origin, with or without carcinoid syndrome, who had failed at least one prior systemic anticancer therapy were the principal eligibility criteria. Concurrent therapy with a somatostatin analog (SSA), evidence of radiologic disease progression prior to study start with no receptor-targeted radio-labelled or liver-directed therapy within 3–4 months, and an Eastern Cooperative Oncology Group (ECOG) performance status score no higher than 1 were also requirements for enrolment. Specified laboratory results for inclusion were a hemoglobin level of ≥9 g/dL, absolute neutrophil count ≥1.5×10^9^/L, platelet count ≥100×10^9^/L, serum potassium within normal limits or correctable with supplements, aspartate and alanine aminotransferases ≤2.5×upper limit of normal (ULN) or ≤5.0×ULN if liver tumor was present, bilirubin ≤1.5×ULN or ≤2×ULN if liver tumor was present, creatinine ≤1.5×ULN or 24-hour clearance ≥50 mL/min, and a negative serum or urine pregnancy test within 48 hours before starting CC-223 for females of childbearing potential. Patients with pancreatic NET, pulmonary carcinoid, high-grade poorly differentiated, or rare variant tumor types were excluded.

**Fig 1:**
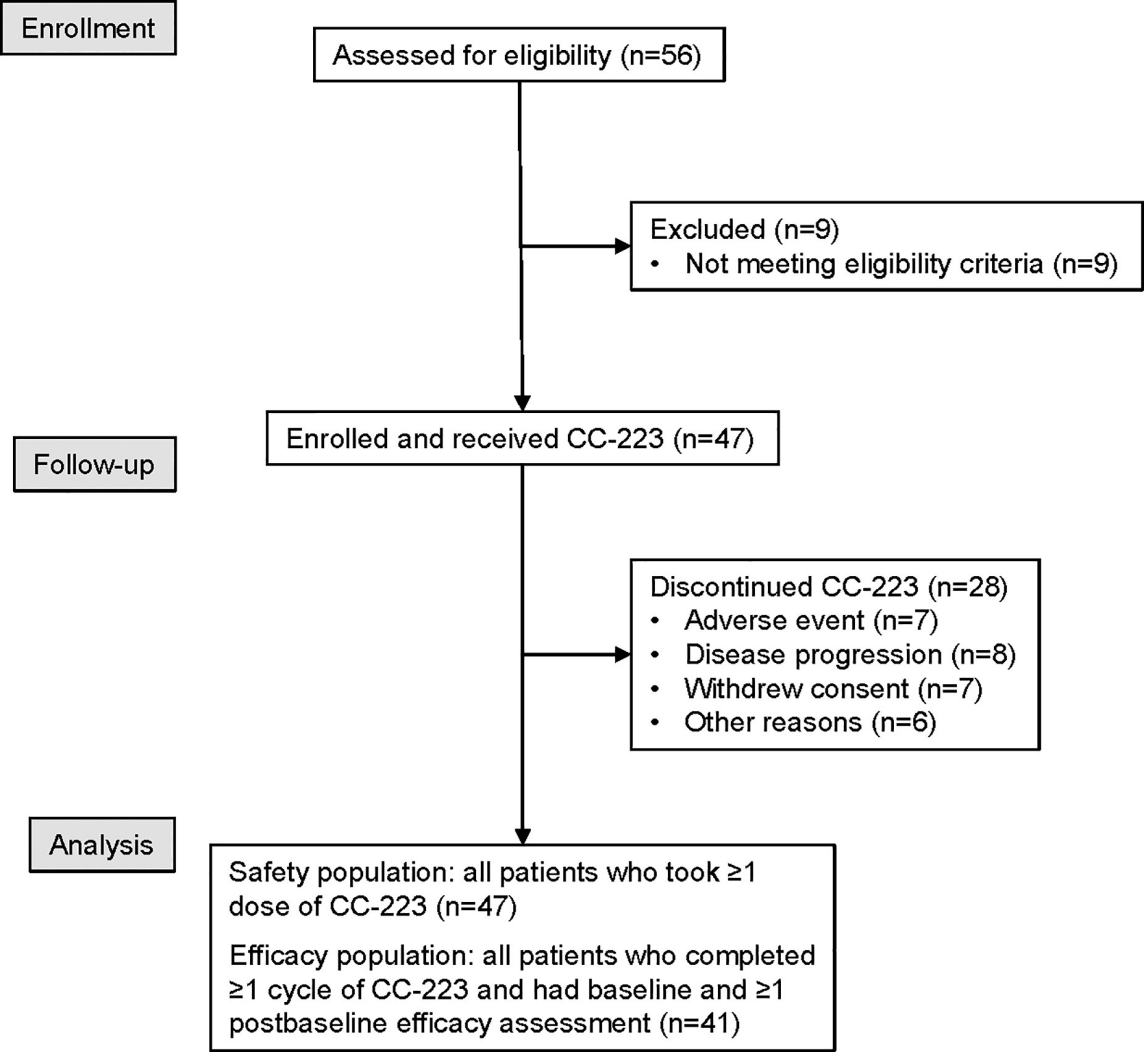
CONSORT diagram.

Using the optimal CC-223 dose of 45 mg established in phase 1 [13], cohort expansion objectives were to determine the tolerability of CC-223, characterize the pharmacokinetic (PK) and pharmacodynamic (biomarker) profiles, and identify a preliminary efficacy signal in seven parallel cohorts of various preselected tumor types; only the NET cohorts are reported here. Patient flow is shown in Fig 1. The study (NCT01177397) was conducted in accordance with the International Conference on Harmonisation (ICH) E6 requirements for Good Clinical Practice, in accordance with the ethical principles outlined in the Declaration of Helsinki and according to all other national and institutional guidelines. The study protocol was approved by the institutional review boards and other necessary oversight committees at each investigational site (ie, Integreview Ethical Review Board, Committee of Human Research Office of Research, Western Institutional Review Board, Mary Crowley Medical Research Center, North Texas Institutional Review Board at Medical City, Western Institutional Review Board, NYU School of Medicine Institutional Review Board, Office of the Human Research Protection Program (OHRPP) UCLA Medical Institutional Review Board, Cedars-Sinai Medical Center Institutional Review Board Office of Research Compliance, Liberty IRB, Inc, Mayo Clinic Institutional Review Board, Comité Autonomico de Ensayos Clinicos deAndalucia, Comité Ético de Investigación Clínica Hospital Clínico Universitario de Salamanca, Comité Consultatif de Protection des Personnes dans la Recherche, Comité de Protection des Personnes Ile de France III and NRES Committee London–Westminster). The protocol and supporting CONSORT checklist are available as supporting information (S1 Checklist and S1 Protocol). The first patient was enrolled into the study on July 18, 2011 and the last patient completed the study on October 25, 2016.

## Procedures

CC-223 capsules were taken orally once daily in continuous 28-day cycles. An initial cohort started CC-223 at a dose of 45 mg; this was the maximum tolerated dose (MTD) established in phase 1 but, after observing a high rate of dose adjustments due to toxicity among patients with NET, a second cohort started CC-223 at 30 mg. To mitigate toxicity, dosage could be adjusted with either short interruptions, dose level reductions, or every-other-day administration. Treatment was permanently discontinued for disease progression, unmanageable toxicity, patient withdrawal of consent, or other reasons specified by the protocol as requiring study withdrawal.

Baseline assessments were completed within 28 days prior to first dose of CC-223. Drug tolerability was continuously evaluated until 28 days after the last dose, using clinical event and laboratory test monitoring comprising a conventional hematologic and coagulation (if taking anti-coagulants) panel; serum electrolytes; fasting blood glucose (this also included daily self-monitoring with finger stick samples for the first month and for longer if necessary), HbA_1c_, insulin, and c-peptide; liver enzymes; serum creatinine; and protein. Others included serum uric acid, amylase and lipase, lipoproteins, thyroid function tests, creatine kinase, and immunoglobulins including T-cell subsets (CD4+ and CD8+). We also performed serial triplicate electrocardiograms (ECGs), periodic left ventricular ejection fraction estimations (echo or multiple gated acquisition [MUGA]), regular physical exams and vital sign measurements (heart rate, blood pressure, respiratory rate, and temperature). Adverse events were coded with Medical Dictionary for Regulatory Activities (MedDRA) version 19.0. Severity of adverse events was classified using the National Cancer Institute Common Terminology Criteria for Adverse Events (CTCAE) version 4.0. A safety review committee provided study oversight.

Tumors were restaged by the principal investigator at each site using computed tomography (CT), positron emission tomography (PET)-CT, or magnetic resonance imaging scans after every 2 cycles through cycle 6, and every 3 cycles thereafter, using Response Evaluation Criteria In Solid Tumors (RECIST) version 1.1 criteria for response [14].

Metabolic changes were assessed by percent change in total of standardized uptake value (SUV) of 2-^18^F-fluoro-2-deoxyglucose (FDG) with PET scanning from baseline to day 15 in cycle 1. Carcinoid symptoms of flushing and diarrhea were documented monthly with a non-validated symptom questionnaire that was completed at each study visit. Patients reported whether they had experienced specific events, and the severity and/or frequency of these events. Symptom improvement was defined as ≥50% reduction in frequency and/or a 1-grade reduction in severity for each from baseline.

Central laboratories conducted serial stimulated blood biomarker assays in monocytes (CD14+) for p4E-BP1 and pPKB (also known as pAKT) for assessment of mTORC1 and mTORC2 inhibition, respectively. Monthly changes from baseline for chromogranin A (CgA), and for other serum hormones if elevated, were assayed locally.

Blood for PK analysis was drawn on day 1 pre-dose and for up to 48 hours post-dose, and then on day 15 up to 8 hours post-dose. Urine on day 1 was collected within 30 minutes prior to dosing and at intervals up to 24 hours post-dose. Samples were assayed for CC-223 and the principal active metabolite (M1) using validated chiral liquid chromatography mass spectrometry. CC-223 PK parameters were calculated using dosing and sample collection times. Non-compartmental PK analysis was performed used WinNonlin Enterprise version 5.2 Model 200 and Model 210 (Certara, Princeton, NJ).

### Outcomes

The primary objectives were to investigate the safety and tolerability and determine the preliminary PK of CC-223 administered orally. Secondary objectives were to characterize the PK of M1, to evaluate the inhibition of mTORC1 and mTORC2 in peripheral blood using p4E-BP1 and pAKT as biomarkers, and to evaluate the preliminary efficacy of CC-223 in terms of tumor response, PFS, and overall survival (OS).

### Statistical analysis

This phase 1/2 study was not powered for inferential statistics and mostly descriptive statistics were used. All patients dosed with CC-223 were included in the safety analysis. The efficacy analysis included all patients completing at least one cycle of drug who had both baseline and one post-baseline tumor efficacy assessment, unless otherwise specified. Categorical baseline variables including tumor type-specific characteristics were summarized using frequency counts and percentage. Continuous demographic and baseline characteristic variables were summarized by descriptive statistics. A Wilcoxon signed rank test was used to analyse change from baseline in serum hormone levels at selected scheduled visits.

Best overall tumor response by investigator assessment was summarized using frequency tabulation. Waterfall plots were used to show best percentage change from baseline in the sum of length (longest diameter) of target lesions.

Relationship between PFS and NET-specific assessments such as serum hormone levels and carcinoid symptoms were investigated. The Kaplan-Meier estimate of median PFS with its two-sided 95% confidence interval [CI] was provided for each baseline category of the selected biomarkers. Kaplan-Meier plots of PFS were created by category. The raw *P* value of the log rank test comparing survival distribution of PFS between categories was also provided.

Approximately 40 evaluable patients with NET were planned for further evaluation of safety and preliminary antitumor activity. Patients could receive CC-223 at the MTD and/or a lower dose level based on the safety, PK, and PD data from the dose escalation phase [13].

Enrollment and tumor response rate monitoring were based on Wald’s sequential analysis [15]. Assuming a target response rate of 20% for the tumor type of interest (NET), based on the sequential design, with 80% power to identify the group with 20% or more response rate at 5% significance level when the response rate is at 10%, the enrollment could be stopped for futility when there was no responder out of up to 14 evaluable patients.

## Results

The study was conducted between July 2011 and October 2016 at 15 investigational sites. From a total of 58 screened patients, 47 met all eligibility criteria. Of those, the first 24 started CC-223 at the MTD of 45 mg/day and the subsequent 23 started at 30 mg/day. Median age was 63 years (min-max: 35–79 years) (Table 1). Men comprised 40% of the population; 92% of patients were white, and 64% had ECOG performance status score of 1 on entry. Most (91%) had received prior SSA therapy and 43% had received additional systemic treatment; no patient received prior mTOR inhibitor therapy; however, one patient received concomitant treatment with everolimus. The majority (57%) had midgut, followed by other GI (23%) tumors, and 53% reported NET-related symptoms of either flushing or diarrhea. Eighteen patients (38%) withdrew from the study due to progression of their disease, 9 (19%) due to withdrawal of consent, 8 (17%) due to adverse events, 2 patients (4%) died, and 10 (21%) discontinued for other reasons.

**Table 1:** Patient demographics and baseline characteristics (n = 47).

**Age**	
Median years (min, max)	63 (35, 79)
≤65 years	26 (55.3)
>65 years, n (%)	21 (44.7)
**Sex, %**	
Males	19 (40.4)
Females	28 (59.6)
**Race, n (%)**	
White	43 (91.5)
Asian	2 (4.3)
Black	1 (2.1)
Other	1 (2.1)
**Primary site, n (%)**	
Midgut	27 (57.4)
Gastrointestinal not specified	11 (23.4)
Rectal	1 (2.1)
Not specified	8 (17.0)
**ECOG PS, n (%)**	
0	17 (36.2)
1	30 (63.8)
**Prior therapy other than somatostatin analogs, n (%)**	
0	38 (80.9)
1	5 (10.6)
2	2 (4.3)
≥3	2 (4.3)
**Carcinoid-related symptoms,**^**a**^ **n (%)**	
Flushing	16 (34.0)
Diarrhea	22 (46.8)
Flushing and diarrhea	13 (27.7)

ECOG PS, Eastern Cooperative Oncology Group performance status.

^a^Two symptoms collected partly retrospectively with a non-validated multi-symptom carcinoid questionnaire.

Median treatment duration was 252 days, or 9 cycles (maximum of 1517 days, or 54 cycles), but only 12 (26%) patients remained on the dose initially assigned. Dose adjustment was frequent; overall, 12 (26%), 19 (40%), and 4 (9%) patients required 1, 2, or more reductions, respectively, with a mean time to first dose reduction of 46 days (range 8–589 days). By dose group, 11 (48%), 7 (30%), and 0 patients in the 30 mg group and 3 (13%), 12 (50%), and 2 (8%) patients in the 45 mg group required 1, 2, or more reductions, respectively. The mean dose overall was 26 mg (min-max: 15 [intermittently]–45 mg [once daily]). Patient exposure information is shown in **S1 Fig**.

Of the 46 (98%) patients who reported at least one treatment-related adverse event (TRAE) by investigator determination, 37 (79%) experienced grade 3 or 4 events. Overall, 8 of 47 patients (17%) experienced one or more serious adverse event related to CC-223. A total of 40 (85%) patients required dose adjustments due to toxicity and 6 (13%) discontinued CC-223 because of toxicity. Most common TRAEs were diarrhea (77%), fatigue (66%), stomatitis (53%), pruritus (40%), rash and nausea (38% each), hyperglycemia (28%), decreased appetite and maculopapular rash (26% each), and asthenia (23%) (**Table 2**). Most frequent grade 3 or higher toxicities were diarrhea (38%), fatigue (21%), and stomatitis (11%); there were no grade 4 or 5 toxicities reported for the most common TEAEs. Diarrhea (9%), decreased appetite (6%), and nausea (4%) were the more common toxicities causing C-223 discontinuation, whereas diarrhea (40%), stomatitis and fatigue (17% each), and increased blood creatinine (13%) required the most dose adjustments. No patient died on study due to treatment-related toxicity.

**Table 2:** Most common treatment-related adverse events in ≥10% of patients (n = 47).

	Any dose (N = 47)		CC-223 30 mg (n = 23)	CC-223 45 mg (n = 24)
Adverse event, n (%)	Any grade, n (%)	Grade 3,^a^ n (%)	Any grade, n (%)	Any grade, n (%)
*Gastrointestinal disorders*				
Diarrhea	36 (76.6)	18 (38.3)	17 (73.9)	19 (79.2)
Stomatitis	25 (53.2)	5 (10.6)	11 (47.8)	14 (58.3)
Nausea	18 (38.3)	1 (2.1)	6 (26.1)	12 (50.0)
Vomiting	7 (14.9)	2 (4.3)	2 (8.7)	5 (20.8)
Dry mouth	7 (14.9)	0	2 (8.7)	5 (20.8)
Abdominal pain	5 (10.6)	1 (2.1)	2 (8.7)	3 (12.5)
*General disorders*				
Fatigue	31 (66.0)	10 (21.3)	17 (73.9)	14 (58.3)
Asthenia	11 (23.4)	3 (6.4)	7 (30.4)	4 (16.7)
Malaise	7 (14.9)	0	3 (13.0)	4 (16.7)
Mucosal inflammation	6 (12.8)	0	3 (13.0)	3 (12.5)
*Investigations*				
Blood creatinine increased	7 (14.9)	1 (2.1)	4 (17.4)	3 (12.5)
AST increased	5 (10.6)	2 (4.3)	3 (13.0)	2 (8.3)
*Metabolism and nutrition disorders*				
Hyperglycemia	13 (27.7)	0	5 (21.7)	8 (33.3)
Decreased appetite	12 (25.5)	1 (2.1)	4 (17.4)	8 (33.3)
Dehydration	8 (17.0)	1 (2.1)	4 (17.4)	4 (16.7)
*Nervous system disorders*				
Dysgeusia	8 (17.0)	0	3 (13.0)	5 (20.8)
*Skin and subcutaneous tissue disorders*				
Pruritus	19 (40.4)	2 (4.3)	7 (30.4)	12 (50.0)
Rash	18 (38.3)	1 (2.1)	6 (26.1)	12 (50.0)
Maculopapular rash	12 (25.5)	3 (6.4)	3 (13.0)	9 (37.5)
Rash macular	6 (12.8)	1 (2.1)	2 (8.7)	4 (16.7)

Adverse events were graded according to Common Terminology Criteria for Adverse Events version 4.0. AST, aspartate aminotransferase.

^a^Treatment-related adverse events were grade 3 only; no grade 4 or grade 5 were reported for these most common toxicities.

The PK results for patients with NET were consistent with results previously reported for phase 1 [13]. The sample size for patients with NET who had serial PK sampling was small (**Table 3**) and included patients who had dose adjustments; both were limitations to these analyses. Overall, regardless of starting dose, CC-223 was rapidly absorbed with maximum plasma concentrations occurring at a median T_max_ of 1.5–3 hours and, for M1, 3–8 hours. CC-223 and M1 exposure (C_max_ and AUC_t_) increased from 30 mg to 45 mg and aligned with dose-proportional increases in exposure observed in the overall dose range evaluated in phase 1 [13]. Significant accumulation of M1 was observed after repeated dosing.

**Table 3:** Pharmacokinetic characteristics for CC-223 and principal metabolite (M1) in patients with NET initially treated with 30 mg or 45 mg CC-223.

Parameter	30 mg/day				45 mg/day	
	Day 1		Day 15		Day 1	
	CC-223	M1	CC-223	M1	CC-223	M1
T_max_^a^ (h)	1.70 (1.5, 3.0, n = 3)	3.12 (3.0, 5.0, n = 3)	1.50 (1.5, 1.5, n = 1)	1.50 (1.5, 1.5, n = 1)	1.52 (1.5, 1.5, n = 2)	6.50 (5.0, 8.0, n = 2)
C_max_ (ng/mL)	133 (34.1, n = 3)	507 (56.3, n = 3)	154 (N/A, n = 1)	486 (N/A, n = 1)	347 (26.9, n = 2)	770 (22.6, n = 2)
AUC_t_ (ng*h/mL)	840 (34.9, n = 3)	6928 (42.5, n = 3)	776 (N/A, n = 1)	6753 (N/A, n = 1)	1796 (10.6, n = 2)	7583 (146.4, n = 2)
AUC_τ_ (ng*h/mL)	819 (34.9, n = 3)	6905 (42.7, n = 3)	776 (N/A, n = 1)	6753 (N/A, n = 1)	1935 (N/A, n = 1)	16115 (N/A, n = 1)
CL/F (L/h)	34.0 (34.2, n = 3)	N/A	N/A	N/A	19.8 (20.1, n = 2)	N/A
Vz/F (L)	290 (58.3, n = 3)	N/A	N/A	N/A	125 (6.9, n = 2)	N/A

Data are presented as geometric mean (%CV, n), except where indicated. AUC_t_, area under the plasma concentration-time curve from time 0 to the last measurable concentration at time t; AUC_τ_, area under the plasma concentration-time curve from time 0 to τ, where τ is the dosing interval; CL/F, apparent total body clearance; C_max_, maximum observed concentration in plasma; CV, percentage coefficient of variation; h, hour; N/A, not applicable; T_max_, time to maximum concentration; Vz/F, apparent volume of distribution.

^a^Median (min, max, n) data are presented.

Across both dose level cohorts in this study, substantial median biomarker inhibition relative to baseline was achieved for pAKT (>70%) and p4E-BP1 (>26%) at 1.5 hours after multiple dosing on day 15 regardless of whether patients started at 45 mg or 30 mg (**Table 4**). Among the 34 of 47 (72%) patients with NET who had evaluable biomarker results, 13 and 21 patients were dosed initially with 45 mg and 30 mg CC-223, respectively. At 45 mg, blood pAKT showed a median inhibition of 83% 1.5 hours after a single dose (day 1) and 76% after multiple doses (day 15) of CC-223. Inhibition of p4E-BP1 was more moderate and showed a median inhibition of 37% 1.5 hours after a single dose and 47% inhibition after multiple doses. At the lower starting dose of 30 mg, weaker inhibition was observed after the first dose for both biomarkers, but the difference became less pronounced by day 15 after repeated dosing and disappeared completely for pAKT. At 30 mg, pAKT showed a median inhibition from baseline of 59% after the first dose and of 79% at day 15 after multiple doses, while p4E-BP1 showed a median inhibition of 9% following a single dose and of 26% after multiple doses. Taken together, these results demonstrate good inhibition of pAKT and less but meaningful 4E-BP1 inhibition regardless of CC-223 dose after repeat dosing at these dose levels. However, as noted above, the majority of patients could not tolerate the starting doses, and doses were reduced for extended periods to levels as low as 15 mg for 45% of patients. The pharmacodynamic effects at lower doses were not evaluated. Due to challenges obtaining optional paired biopsies in this population, the effects of CC-223 in tumor tissue could not be evaluated.

**Table 4:** Regulation of pAKT and p4E-BP1 in monocytes. Inhibition (percent change from baseline) for pAKT and p4E-BP1 in monocytes of patients initially dosed with 45 mg or 30 mg CC-223.

	pAKT						p4E-PB1					
	30 mg/day			45 mg/day			30 mg/day			45 mg/day		
	C1D1 1.5 h	C1D15 0 h	C1D15 1.5 h	C1D1 1.5 h	C1D15 0 h	C1D15 1.5 h	C1D1 1.5 h	C1D15 0 h	C1D15 1.5 h	C1D1 1.5 h	C1D15 0 h	C1D15 1.5 h
n	21	15	15	13	9	8	21	15	15	13	9	8
Median	–59	–63	–79	–83	–43	–76	–9	–18	–26	–37	–27	–47
Mean	–56	–45	–65	–79	–40	–78	–9	–13	–30	–31	–29	–41
SD	25	42	31	13	43	9	20	29	30	35	35	29
Min	–94	–94	–93	–99	–93	–89	–55	–65	–79	–85	–79	–68
Max	–7	42	23	–44	44	–67	26	35	26	46	27	23

C, cycle; D, day; h, hour; pAKT, protein kinase B; p4E-BP1, 4E-binding protein 1; SD, standard deviation.

**Fig 2:**
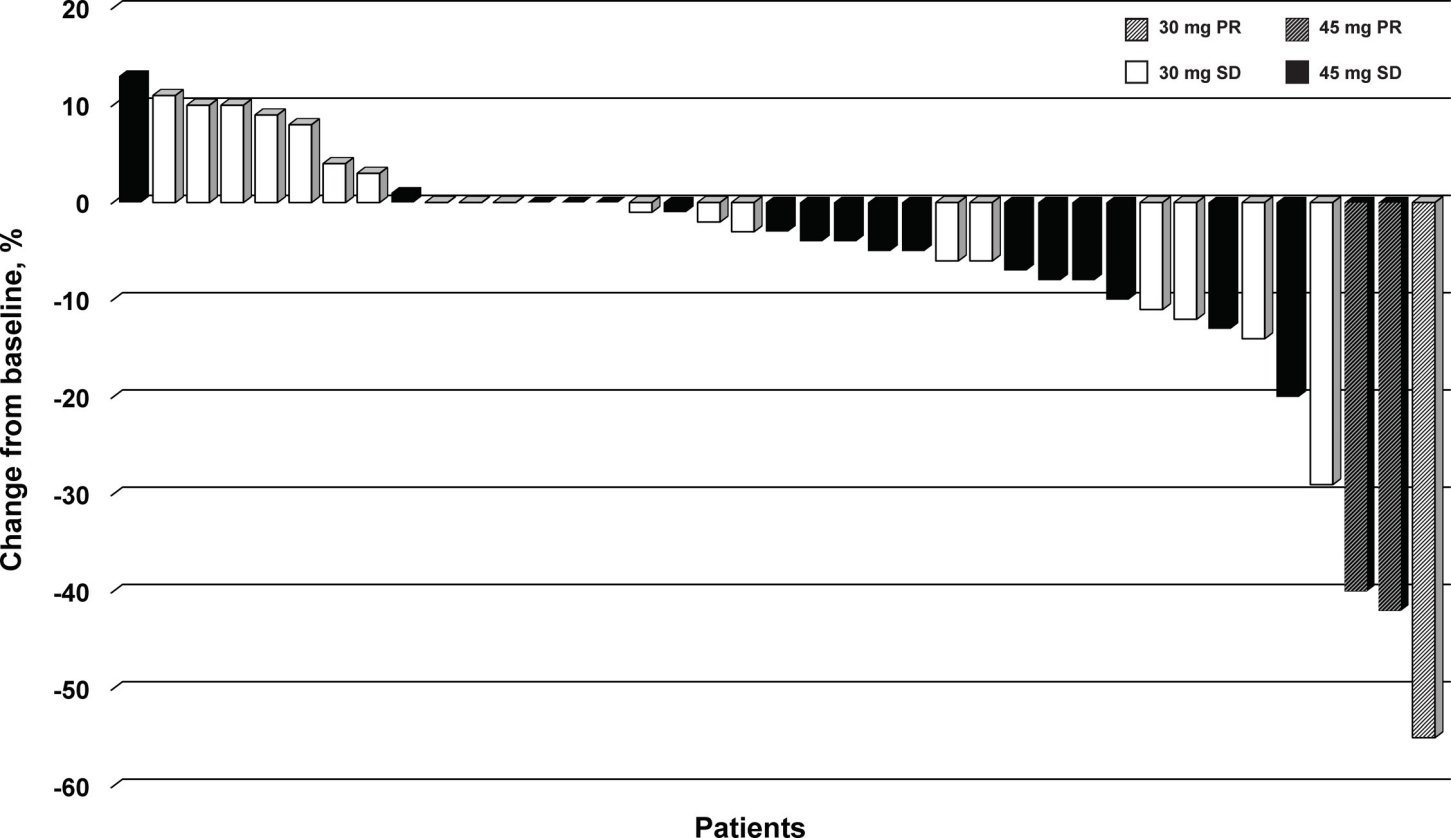
Waterfall plot of tumor response according to RECIST version 1.1 in patients treated with CC-223, by starting dose. Best percent change from baseline in the sum of the longest diameters of target lesions (n = 39). PR, partial response; RECIST, Response Evaluation Criteria In Solid Tumors; SD, stable disease.

Mean percent decrease from baseline to day 15 of treatment in [^18^F]-FDG PET SUV in 31 patients was 32.0% (standard deviation 35.2%; *P*<0.0001, Wilcoxon signed rank test). Twenty-one patients (44.7%) exhibited at least a 15% reduction in uptake and 18 (38.3%) had at least a 25% reduction in SUV. These metabolic changes did not correlate strongly with subsequent tumor response outcomes using RECIST criteria (Fig 2).

**Fig 3:**
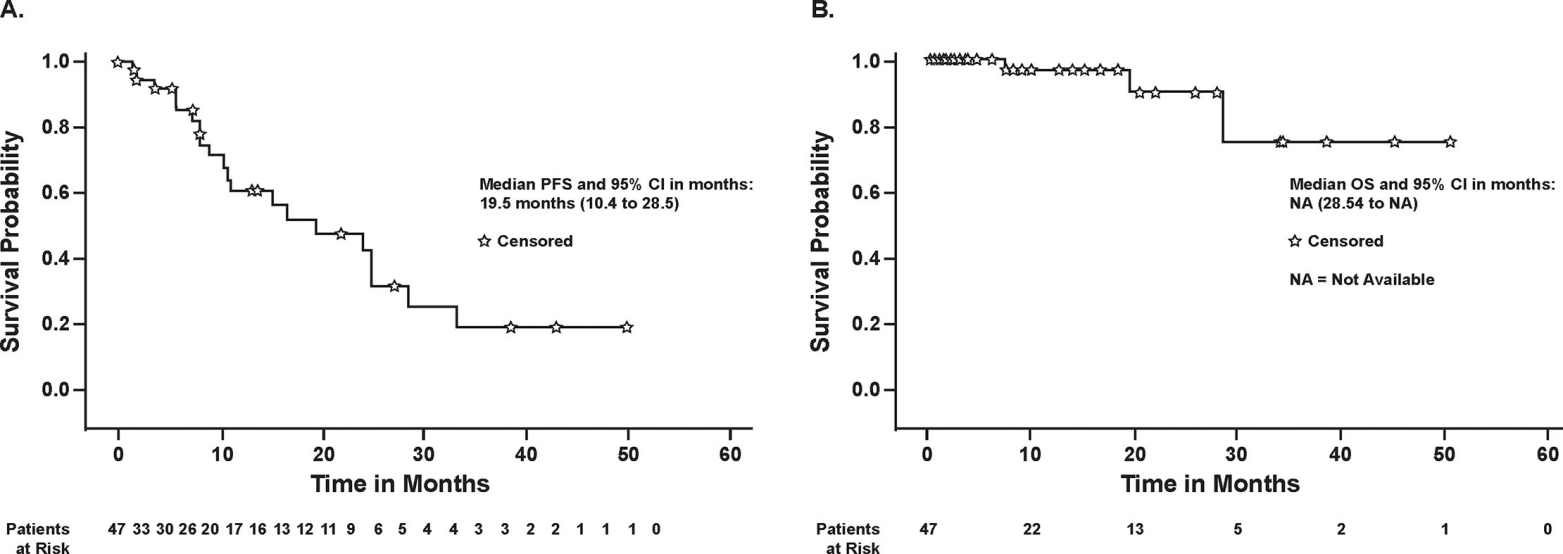
Progression-free survival (A) and overall survival (B) for the treated population. ★, censored; CI, confidence interval; NA, not assessable; OS, overall survival; PFS, progression-free survival.

Of the 47 patients, 41 completed at least one cycle of CC-223, had at least one restaging, and were thus evaluable for efficacy. By investigator assessment, no patient had a complete response (CR), 3 patients (7.3%) had a partial response (PR), 34 (82.9%) showed stable disease (SD), 1 (2.4%) had progressive disease (PD), and for 3 (7.3%) the assessment was not done. Thus, the objective response rate (CR+PR) was 7.3% (95% CI 1.5–19.9%) and the disease control rate (CR+PR+SD) was 90.2% (95% CI 76.9–97.3%). It is noteworthy that tumor shrinkage of any magnitude relative to baseline was observed in 73.2% patients (95% CI 57.1–85.8%) (Fig 2). Duration of response for the three patients with PR was 125, 253+ (no PD reported), and 401 days. The median duration of SD was long and lasted 297 days (min 50 days, max 1519 days). Duration of SD was longer than a year in 17 patients. Median PFS for the treated population was 19.5 months (95% CI 10.4–28.5 months) with rates at 6 and 10 months of 85% and 64%, respectively; median OS was not assessable (OS rate at 48 weeks was 0.97) (Fig 3).

On enrollment, carcinoid-related flushing and diarrhea were reported in 16 (34%) and 22 (47%) patients, respectively, with both reported in 13 patients (28%). Symptom reduction at any time during the study was observed for flushing in 8 (50%) patients, for diarrhea in 9 (41%) patients, and for both symptoms in 5 (39%) patients. An *ad hoc* exploratory analysis suggested that patients with carcinoid symptom improvement had a higher PFS than patients without improvement (median survival [95% CI] 108.4 [72.6–not estimable] weeks *vs* 48.1 [35.6–104.4] weeks).

Among the 35 patients with elevated baseline levels in at least one hormone, a ≥50% decline from baseline was reported for CgA (7 patients), gastrin (6 patients), glucagon (3 patients), and serotonin (2 patients). Sample sizes were small and hormones fluctuated considerably over time; none of the changes from baseline were statistically significant except for CgA (cycle 21 and end of treatment; *P*≤0.039 [log rank text] for each cycle) and serotonin in some later cycles (cycles 11, 13, 15, 17, 19, 21, 23, 25, and end of treatment; *P*≤0.037 [log rank test] for each cycle). One responder had a >50% decline in CgA, and one responder had a >50% decline in gastrin. Too few patients with any elevated baseline levels had sufficient tumor response data for meaningful correlative analysis.

## Discussion

This study provides encouraging first results on response of patients with well-differentiated non-pancreatic GI NET to a TOR kinase inhibitor in combination with an SSA. The extended times on treatment and durable PFS were notable (median PFS of 19.5 months for CC-223), considering that previously published data for everolimus reported a median PFS of 11.0 months [3, 4]. These findings support preclinical data indicating that dual inhibition by CC-223 may have advantages over everolimus, for example in everolimus-refractory patients [12]. The frequency of carcinoid symptom reduction, which started early in patients who were refractory to an SSA alone, was also of interest. Since symptom monitoring using a non-validated questionnaire was added partway through the study in response to patients spontaneously reporting early-onset marked relief in both flushing and diarrhea, further evaluation of this observation is needed.

The selection of an optimal dose of CC-223 at the conclusion of the dose-finding phase of the study was based on considerations of drug tolerability, magnitude and duration of mTORC1 and mTORC2 biomarker inhibition, and preliminary efficacy observed in a mixed tumor population [13]. The 45 mg CC-223 once daily was not well-tolerated in the NET cohort. Dose reductions were also reported for >50% of patients in tumor-specific cohorts of non-small cell lung cancer and hepatocellular carcinoma dosed at 45 mg [16]. Most dose reductions occurred during the first 1–2 cycles of treatment. Additionally, while most adverse events occurred at lower rates at the 30 mg/day dose in patients with NET (**Table 2**), the 30-mg cohort also required frequent dose adjustments. Longer treatment duration was achieved at significantly lower doses for both cohorts. This may be partly explained by the relatively less aggressive disease characteristics of NET and the reluctance of these highly functioning patients to tolerate the inconvenience of adverse effects associated with their cancer treatment. Further consideration is needed to determine whether it is better to start treatment at 30 mg/day (ie, close to the conventional mean dose administered [26 mg]) or to start at a much lower dose and titrate up to an optimally tolerable level in order to improve compliance and quality of life. To minimize toxicity and maintain compliance, a number of patients required dose reductions to as low as 15 mg per day, with 3 patients at this dose every other day. At the 15 mg dose level, target biomarker inhibition would be predicted (not tested) to be modest, yet these patients benefitted from durable tumor shrinkage for extended periods of time. These findings suggest it may be appropriate to re-evaluate the traditional practice in dose-finding studies of dosing close to MTD for cohort expansion, and instead consider using the dose achieving maximal biomarker inhibition. This is particularly relevant for oral, daily administered drugs where cumulative toxicities often occur beyond the “classical” four-week dose-limiting toxicity period.

The PET SUV findings approximately 2 weeks into therapy showed a statistically significant reduction in glucose uptake from baseline. However, among patients who achieved at least some metabolic response (at least a 15% reduction from baseline) there were no strong correlations between early metabolic improvements and subsequent tumor response assessed by RECIST (Fig 2). This outcome may have been confounded by CC-223–related hyperglycemia. Furthermore, it was anticipated that well-differentiated NET would have generally low FDG-PET uptake because of its low metabolic rate, as was confirmed in this cohort. Reductions in CgA and other abnormal serum hormones were generally not statistically significant, but patients with carcinoid symptom improvement showed a numerically higher PFS probability than patients without symptom improvement (median PFS 108 weeks *vs* 48 weeks). Correlations between carcinoid symptom improvement and reduction in baseline serum hormone levels were inconclusive, possibly because of the small sample sizes and the multiplicity of analyses (S1 Fig).

The safety profile of CC-223 appears to be comparable with the previously characterized class of approved mTOR inhibitors [3, 17–19], notwithstanding the increased incidence and severity of certain TRAEs. However, toxicity generally emerged early into treatment and was well-managed either by aggressive dose adjustments or standard treatment interventions. The commonly experienced on-target class effect of hyperglycemia correlated with drug exposure and was managed effectively with dose-reduction or with hypoglycemic agents [20]. No late-onset new toxicity was identified even after extended treatment with CC-223. Overall, neither unexpected nor new major safety signals were identified for CC-223 compared with currently available mTOR inhibitors.

In conclusion, treatment with CC-223 showed encouraging preliminary evidence of anti-tumor activity in patients with advanced metastatic non-pancreatic GI NET. The durable tumor responses, coupled with meaningful symptom relief in patients reporting carcinoid symptoms, support further evaluation of CC-223 in this patient population. Dual mTORC1 and mTORC2 kinase inhibitors may have advantages over everolimus, for example in everolimus-refractory patients, as has been shown pre-clinically [12]. In addition, CC-223 efficacy may be enhanced by combination with other cancer therapies, which will be worthy to explore.

## Supporting information

S1 File CONSORT checklist. (PDF)

S2 File Trial protocol. (PDF)

S3 File Supporting Information. (DOCX)

S1 Fig **CC-223 dose adjustments and duration, best overall RECIST version 1.1 and target lesion response, and carcinoid symptom improvement, for patients started at 45 mg/day (A) and 30 mg/day (B) CC-223.** Cycle, total treatment cycles completed; RECIST, best overall response; TL change (%), best target lesion change from baseline. ★, carcinoid symptomatic improvement; NE, not evaluable; PD, progressive disease; PR, partial response; RECIST, Response Evaluation Criteria In Solid Tumors; SD, stable disease.
(TIF)

## References

[1] Yao JC, Hassan M, Phan A, Dagohoy C, Leary C, Mares JE, et al One hundred years after "carcinoid": epidemiology of and prognostic factors for neuroendocrine tumors in 35,825 cases in the United States. J Clin Oncol. 2008;26(18):3063–72. 10.1200/JCO.2007.15.4377 . 18565894

[2] National Cancer Institute. SEER Cancer Statistics Review 1975–2016 [Accessed May 3, 2019]. Available from: https://seer.cancer.gov/csr/1975_2016/results_merged/topic_prevcounts.pdf.

[3] Yao JC, Fazio N, Singh S, Buzzoni R, Carnaghi C, Wolin E, et al Everolimus for the treatment of advanced, non-functional neuroendocrine tumours of the lung or gastrointestinal tract (RADIANT-4): a randomised, placebo-controlled, phase 3 study. Lancet. 2016;387(10022):968–77. 10.1016/S0140-6736(15)00817-X 26703889 PMC6063317

[4] Yao JC, Shah MH, Ito T, Bohas CL, Wolin EM, Van Cutsem E, et al Everolimus for advanced pancreatic neuroendocrine tumors. The New England journal of medicine. 2011;364(6):514–23. Epub 2011/02/11. 10.1056/NEJMoa1009290 21306238 PMC4208619

[5] Kim JO, Kim KH, Song IS, Cheon KS, Kim OH, Lee SC, et al Potentiation of the anticancer effects of everolimus using a dual mTORC1/2 inhibitor in hepatocellular carcinoma cells. Oncotarget. 2017;8(2):2936–48. Epub 2016/12/10. 10.18632/oncotarget.13808 27935857 PMC5356853

[6] Chan JY, Choudhury Y, Tan MH. Doubling Down on mTOR Inhibition: Harnessing ZEBRA for Insights. Eur Urol. 2016;69(3):457–9. Epub 2015/10/16. 10.1016/j.eururo.2015.09.047 . 26463319

[7] Freitag H, Christen F, Lewens F, Grass I, Briest F, Iwaszkiewicz S, et al Inhibition of mTOR's Catalytic Site by PKI-587 Is a Promising Therapeutic Option for Gastroenteropancreatic Neuroendocrine Tumor Disease. Neuroendocrinology. 2017;105(1):90–104. Epub 2016/08/12. 10.1159/000448843 27513674 PMC5475233

[8] Kannan A, Lin Z, Shao Q, Zhao S, Fang B, Moreno MA, et al Dual mTOR inhibitor MLN0128 suppresses Merkel cell carcinoma (MCC) xenograft tumor growth. Oncotarget. 2016;7(6):6576–92. Epub 2015/11/05. 10.18632/oncotarget.5878 26536665 PMC4872734

[9] Musa F, Alard A, David-West G, Curtin JP, Blank SV, Schneider RJ. Dual mTORC1/2 Inhibition as a Novel Strategy for the Resensitization and Treatment of Platinum-Resistant Ovarian Cancer. Mol Cancer Ther. 2016;15(7):1557–67. 10.1158/1535-7163.MCT-15-0926 27196780 PMC5323079

[10] Zou Z, Chen J, Yang J, Bai X. Targeted Inhibition of Rictor/mTORC2 in Cancer Treatment: A New Era after Rapamycin. Current cancer drug targets. 2016;16(4):288–304. Epub 2015/11/14. . 26563881 10.2174/1568009616666151113120830

[11] Mortensen DS, Fultz KE, Xu S, Xu W, Packard G, Khambatta G, et al CC-223, a Potent and Selective Inhibitor of mTOR Kinase: In Vitro and In Vivo Characterization. Mol Cancer Ther. 2015;14(6):1295–305. Epub 2015/04/10. 10.1158/1535-7163.MCT-14-1052 . 25855786

[12] Orr-Asman MA, Chu Z, Jiang M, Worley M, LaSance K, Koch SE, et al mTOR Kinase Inhibition Effectively Decreases Progression of a Subset of Neuroendocrine Tumors that Progress on Rapalog Therapy and Delays Cardiac Impairment. Mol Cancer Ther. 2017;16(11):2432–41. Epub 2017/09/03. 10.1158/1535-7163.MCT-17-0058 . 28864682

[13] Bendell JC, Kelley RK, Shih KC, Grabowsky JA, Bergsland E, Jones S, et al A phase I dose-escalation study to assess safety, tolerability, pharmacokinetics, and preliminary efficacy of the dual mTORC1/mTORC2 kinase inhibitor CC-223 in patients with advanced solid tumors or multiple myeloma. Cancer. 2015;121(19):3481–90. 10.1002/cncr.29422 26177599 PMC4832308

[14] Eisenhauer EA, Therasse P, Bogaerts J, Schwartz LH, Sargent D, Ford R, et al New response evaluation criteria in solid tumours: revised RECIST guideline (version 1.1). Eur J Cancer. 2009;45(2):228–47. 10.1016/j.ejca.2008.10.026 . 19097774

[15] Wald A. Sequential tests of statistical hypotheses. Ann Math Statist. 1945;16(2):117–86.

[16] Varga A, Mita MM, Wu JJ, Nemunaitis JJ, Cloughesy TF, Mischel PS, et al Phase I expansion trial of an oral TORC1/TORC2 inhibitor (CC-223) in advanced solid tumors. Journal of Clinical Oncology. 2013;31(15_suppl):2606-. 10.1200/jco.2013.31.15_suppl.2606

[17] Faggiano A, Malandrino P, Modica R, Agrimi D, Aversano M, Bassi V, et al Efficacy and Safety of Everolimus in Extrapancreatic Neuroendocrine Tumor: A Comprehensive Review of Literature. Oncologist. 2016;21(7):875–86. 10.1634/theoncologist.2015-0420 27053503 PMC4943387

[18] Kaplan B, Qazi Y, Wellen JR. Strategies for the management of adverse events associated with mTOR inhibitors. Transplant Rev (Orlando). 2014;28(3):126–33. 10.1016/j.trre.2014.03.002 . 24685370

[19] Su DW, Mita M, Mita AC. The Clinical Pharmacology and Toxicity Profile of Rapalogs. 1 ed Mita M, Mita A, Rowinsky EK, editors: Springer-Verlag Paris; 2016.

[20] Goldman JW, Mendenhall MA, Rettinger SR. Hyperglycemia Associated With Targeted Oncologic Treatment: Mechanisms and Management. Oncologist. 2016;21(11):1326–36 10.1634/theoncologist.2015-0519 27473045 PMC5189614

