# Supplementary material for: A phase 2 study of an oral mTORC1/mTORC2 kinase inhibitor (CC-223) for non-pancreatic neuroendocrine tumors with or without carcinoid symptoms
Source: PLoS One. 2019 Sep 17;14(9):e0221994. doi: 10.1371/journal.pone.0221994 (PMC6748410; doi:10.1371/journal.pone.0221994)
Supplement: S2 File — (PDF) [file pone.0221994.s002.pdf]

A PHASE 1/2, MULTI-CENTER, OPEN-LABEL, DOSE FINDING STUDY TO ASSESS THE SAFETY, TOLERABILITY, PHARMACOKINETICS AND PRELIMINARY EFFICACY OF THE MTOR KINASE INHIBITOR CC-223 ADMINISTERED ORALLY TO SUBJECTS WITH ADVANCED SOLID TUMORS, NON-HODGKIN LYMPHOMA OR MULTIPLE MYELOMA

**INVESTIGATIONAL PRODUCT (IP):**	**CC-223**
**PROTOCOL NUMBER:**	**CC-223-ST-001**
**ORIGINAL DATE:**	**12 April 2010**
**AMENDMENT No. 1 DATE Final:**	**29 June 2010**
**Amendment No. 2 DATE FINAL:**	**15 September 2010**
**Amendment No. 3 DATE FINAL**	**16 November 2010**
**Amendment No. 4 DATE FINAL**	**26 January 2011**
**Amendment No. 5 DATE FINAL**	**5 May 2011**
**Amendment No. 6 DATE FINAL**	**4 October 2011**
**Amendment No. 7 DATE FINAL**	**16 April 2012**
**Amendment No. 8 DATE FINAL**	**18 July 2012**
**Amendment No. 9 DATE FINAL**	**17 January 2013**
**Amendment No. 10 DATE FINAL**	**15 December 2014**
**EudraCT NUMBER:**	**2010-022442-25**
**IND NUMBER:**	**108204**
**SPONSOR NAME / ADDRESS**	**Celgene Corporation<br>86 Morris Avenue<br>Summit, NJ 07901**

EDMS Doc. Number: 20012337 - 16445273

TABLE OF CONTENTS

TITLE PAGE 1

TABLE OF CONTENTS 10

LIST OF ABBREVIATIONS AND DEFINITIONS OF TERMS 16

2. STUDY OBJECTIVES AND ENDPOINTS 27

2.1. Primary Objective and Endpoints 27

2.2. Secondary Objectives and Endpoints 28

3. OVERALL STUDY DESIGN 30

3.1. Study Design 30

3.2. Study Design Rationale 30

3.3. Study Duration 32

4. TABLE OF EVENTS 33

5. PROCEDURES 40

5.1. Screening (Parts A and B) 40

5.2. During Treatment 42

5.3. Post-Treatment Assessments 48

6. STUDY POPULATION 51

6.1. Number of Subjects and Sites 51

6.2. Inclusion Criteria 51

6.3. Exclusion Criteria 54

7. DESCRIPTION OF STUDY TREATMENTS 56

7.1. Description of Investigational Product(s) 56

7.1.1. Physical properties 56

7.1.2. Formulation 56

EDMS Doc. Number: 20012337 - 16445273

7.1.3. Storage Conditions 56
7.2. Treatment Administration and Schedule 56
7.2.1. Definition of a Treatment Cycle 56
7.2.2. Starting Dose Level for Cycle 1 57
7.2.3. Dose Escalation Levels 57
7.2.4. Definition of an Evaluable Subject..57
7.2.5. Definition of Non-tolerated dose (NTD) 58
7.2.6. Definition of Maximum Tolerated Dose (MTD) 58
7.2.7. Permitted Study Drug Adjustments..59
7.2.8. Treatment Delay 61
7.3. Method of Treatment Assignment..61
7.4. Packaging and Labeling..61
7.5. Investigational Product Accountability and Disposal 61
7.6. Investigational Product Compliance...61

9. STATISTICAL ANALYSES..66
9.1. Overview...66
9.2. Study Population Definitions 66
9.3. Sample Size and Power Considerations..66
9.4. Background and Demographic Characteristics 67
9.5. Subject Disposition...67
9.6. Efficacy Analysis..67
9.7. Safety Analysis..68
9.8. Interim Analysis 68
9.9. Assessment of Pharmacokinetics 68
9.10. Assessment of Pharmacodynamics...69

EDMS Doc. Number: 20012337 - 16445273

9.11. Evaluation of Potential Predictive Biomarkers 69
9.12. Methods and Timing of PK/PD Sampling 69
10. ADVERSE EVENTS 70
10.1. Monitoring, Recording and Reporting of Adverse Events 70
10.2. Evaluation of Adverse Events 70
10.2.1. Seriousness 70
10.2.2. Severity 71
10.2.3. Causality 72
10.2.4. Duration 72
10.2.5. Action Taken 72
10.3. Abnormal Laboratory Values 72
10.4. Pregnancy 73
10.4.1. Females of Childbearing Potential: 73
10.4.2. Male Subjects 73
10.5. Immediate Reporting of Serious Adverse Events 74
10.5.1. Safety Queries 74
10.6. Expedited Reporting of Adverse Events 74
10.7. Celgene Drug Safety Contact Information: 75
11. DISCONTINUATIONS 76

EDMS Doc. Number: 20012337 - 16445273

17. REFERENCES 85

EDMS Doc. Number: 20012337 - 16445273

LIST OF ABBREVIATIONS AND DEFINITIONS OF TERMS

Table 1: Abbreviations and Specialist Terms

Abbreviation or specialist term	Explanation
AE	Adverse Event
Ae	Urinary Excretion (of CC-223 and/or M1)
AFP	Alphafetoprotein
EIAED	Enzyme-Inducing Anti-Epileptic Drug
ANC	Absolute Neutrophil Count
ALT	Alanine Aminotransferase
AST	Aspartate Aminotransferase
AUC	Area Under the Concentration-Time Curve
$AUC_{(24)}$	Area Under the Curve from 0 to 24 Hours
BID	Bis in Die (twice a day)
BM	Bone Marrow
BUN	Blood Urea Nitrogen
CBC	Complete Blood Count
CK	Creatine Kinase
CL_r	Renal Clearance
CL/F	Apparent Total Body Clearance
C_{max}	Maximum Observed Concentration in Plasma
CNS	Central Nervous System
CYP	Cytochrome P450
eCRF	Electronic Case Report Form
CR	Complete Response
CRO	Contract Research Organization
CT	Computed Tomography
CTCAE	Common Terminology Criteria for Adverse Events
CV%	Coefficient of Variation
d	Day
DCR	Disease Control Rate
DLBCL	Diffuse Large B-Cell Lymphoma
DLT	Dose-Limiting Toxicity

EDMS Doc. Number: 20012337 - 16445273

Table 1: Abbreviations and Specialist Terms (Continued)

Abbreviation or specialist term	Explanation
DNA	Deoxyribonucleic Acid
EC	Ethics Committee
ECG	Electrocardiogram
ECHO	Echocardiogram
ECOG PS	Eastern Cooperative Oncology Group Performance Status
EIAED	Enzyme Inducing Anti-Epileptic Drug
EMA	European Medicines Agency
EORTC	European Organisation for Research and Treatment of Cancer
EU	European Union
[^{18}F] FDG PET	2-^{18}F-Fluoro-2-Deoxyglucose-Positron Emission Tomography
FFPE	Formalin-fixed, paraffin embedded (tumor tissue)
FLAIR	Fluid-Attenuated Inversion Recovery
fT4	Free Thyroxine
GBM	Glioblastoma Multiforme
GCP	Good Clinical Practice
G-CSF	Granulocyte Colony Stimulating Factor
GI_{50}	Fifty Percent Growth Inhibition Concentration
GLP	Good Laboratory Practices
GM-CSF	Granulocyte-Macrophage Colony Stimulating Factor
HbA1c	Glycated Hemoglobin
Anti-HBc	Antibody to Hepatitis B Core Antigen
Anti-HCV	Antibody to Hepatitis C Virus
HBsAg	Hepatitis B Surface Antigen
HCC	Hepatocellular Carcinoma
HBV	Hepatitis B Virus
Hct	Hematocrit
HCV	Hepatitis C Virus

EDMS Doc. Number: 20012337 - 16445273

Table 1: Abbreviations and Specialist Terms (Continued)

Abbreviation or specialist term	Explanation
HDPE	High Density Polyethylene
HED	Human Equivalent Dose
hERG	Human Ether-à-go-go-Related Gene
Hgb	Hemoglobin
HRPBC	Hormone receptor positive breast cancer
HNSTD	Highest Non-Severely Toxic Dose
Hr	Hour(s)
IC_{50}	Concentration for 50% Inhibition of Response
ICH	International Conference on Harmonization
IEP	Immunoelectrophoresis
INR	International Normalized Ratio
IP	Investigational Product
IRB	Institutional Review Board
IRS1	Insulin Receptor Substrate 1
IURC	International Uniform Response Criteria (for MM)
IWC	International Workshop Criteria
IV	Intravenous
LC-MS/MS	Liquid Chromatography-Mass Spectrometry Methods
LD	Longest Diameter
LDH	Lactate Dehydrogenase
LFTs	Liver Function Tests
LVEF	Left Ventricular Ejection Fraction
M1	Metabolite (CC-0483131) of CC-223
MCH	Mean Corpuscular Hemoglobin
MCV	Mean Corpuscular Volume
MCHC	Mean Corpuscular Hemoglobin Concentration
MedDRA	Medical Dictionary for Regulatory Activities
Min	Minute
MM	Multiple Myeloma
MRI	Magnetic Resonance Imaging

EDMS Doc. Number: 20012337 - 16445273

Table 1: Abbreviations and Specialist Terms (Continued)

Abbreviation or specialist term	Explanation
MTD	Maximum Tolerated Dose
mTOR	Mammalian Target of Rapamycin
mTORC1/2	Mammalian Target of Rapamycin Complex 1/2
MUGA	Multiple Gated Acquisition Scan
NCI	National Cancer Institute
NET	Neuroendocrine Tumor (of Non-Pancreatic Origin)
NSCLC	Non-Small Cell Lung Cancer
Nocte	At Night
NHL	Non-Hodgkin Lymphoma
NTD	Non-Tolerated Dose
PD	Pharmacodynamic, or Progressive Disease (for tumor response)
PFS	Progression Free Survival
PI3K	Phosphoinositide 3-Kinase
PK	Pharmacokinetic(s)
Plt	Platelet(s)
PO	Per Os (by mouth, orally)
PR	Partial Response
PT	Prothrombin Time
PTEN	Phosphatase and Tensin Homology
PTT	Partial Thromboplastin Time
QD	Quaque Die (once daily)
RANO	Responses Assessment for Neuro-Oncology Working Group for GBM
RBC	Red Blood Cell (count)
RECIST	Response Evaluation Criteria in Solid Tumors
RR	Response Rate
SAE	Serious Adverse Event
SCID	Severe Combined Immune Deficient
SD	Stable Disease
SOP	Standard Operating Procedure(s)
SPD	Sum of the Products of Perpendicular Diameters
SPEP	Serum Protein Electrophoresis

EDMS Doc. Number: 20012337 - 16445273

Table 1: Abbreviations and Specialist Terms (Continued)

Abbreviation or specialist term	Explanation
SRC	Safety Review Committee
STD10	Severely Toxic Dose in 10% of the Animals Tested
$T_{1/2}$	Terminal Half-Life
Tmax	Time to Maximum Concentration
TPR	Time Point Responses
TSC2	Tuberous Sclerosis Complex 2
TSH	Thyroid Stimulating Hormone
ULN	Upper Limit of Normal
UPEP	Urine Protein Electrophoresis
US	United States
Vz/F	Apparent Volume of Distribution
WBC	White Blood Cell (count)

EDMS Doc. Number: 20012337 - 16445273

2. STUDY OBJECTIVES AND ENDPOINTS

Study CC-223-ST-001 is a first-in-man trial in which CC-223 will be administered to subjects with advanced solid tumors, non-Hodgkin lymphoma (NHL), or multiple myeloma (MM) who are resistant to or have relapsed after standard therapy and for whom no standard therapy exists. Study CC-223-ST-001 is designed as a Phase 1/2 trial consisting of two parts: dose escalation (Part A) and dose expansion (Part B).

In Part A, subjects will receive single and multiple ascending doses of CC-223 in cohorts to measure PK and establish the MTD. Part A, the dose escalation phase, will be followed by Part B, the dose expansion phase in which approximately 230 (preselected tumor types in groups of up to 40) evaluable subjects will be recruited for further evaluation of safety and preliminary antitumor activity. In Part B, subjects may receive CC-223 at the MTD and/or a lower dose level, based on the safety, PK and PD data from Part A.

2.1. Primary Objective and Endpoints

Objectives	Endpoints
To determine the safety and tolerability of CC-223 when administered orally and to define the non-tolerated dose (NTD) and the maximum tolerated dose (MTD).	DLTs, NTD and MTD will be evaluated using the NCI CTCAE criteria Version 4.0.
To determine the preliminary pharmacokinetics (PK) of CC-223 following both single and multiple oral dosing of CC-223.	C_{max}, AUC, t_{max}, $T_{1/2}$, CL/F and Vz/F of CC-223 after single and multiple doses.

EDMS Doc. Number: 20012337 - 16445273

2.2. Secondary Objectives and Endpoints

Objectives	Endpoints
To evaluate the extent of inhibition of phosphorylation of S6RP (Ser235/236 and/or Ser240/244) and/or 4EB-P1 (Thr37/46) for mTORC1 activity and AKT (Ser473) and/or other relevant biomarkers for mTORC2 activity in peripheral blood samples and tumor biopsies following treatment with CC-223.	Levels of phosphorylation of S6RP, 4EB-P1, AKT and/or other relevant biomarkers in circulating blood cells and tumor biopsies.
To provide information on the preliminary efficacy of CC-223.	Response rate of each tumor type using tumor-specific response criteria.
To characterize the PK of M1 following oral dosing of CC-223.	C_{max}, AUC, t_{max}, $T_{1/2}$, of M1.

3. OVERALL STUDY DESIGN

3.1. Study Design

Study CC-223-ST-001 is a first-in-man trial in which CC-223 will be administered orally to subjects with advanced solid tumors, NHL or MM. The study is designed as a Phase 1/2 trial consisting of two parts: dose escalation (Part A) and dose expansion (Part B).

In Part A, subjects will receive single and multiple ascending dose levels of CC-223 to measure pharmacokinetics (PK) and to identify the maximum tolerated dose (MTD). A modified accelerated titration design (Simon, 1997) will be used to establish initial toxicity. During the accelerated course, initial cohorts of one subject will be given CC-223 at dose increments of 100% until the first instance of first-course grade 2 or higher toxicity, at which point the accelerated part will be terminated, and this particular cohort will be expanded to 6 subjects. Subsequently, a standard escalation dosing schedule with approximately 50% dose increments and 6 subjects per cohort will be initiated in order to establish the non-tolerated dose (NTD) and MTD. Smaller increments and additional subjects within a dose cohort may also be evaluated.

A dose will be considered to be non-tolerated if 2 evaluable subjects in a dose cohort experience DLT. When a NTD is defined, dose escalation will be stopped. The MTD will be defined as the last dose tested below the NTD with 0 or 1 out of 6 evaluable subjects experiencing DLT during Cycle 1. An intermediate dose (ie, one between the NTD and the last dose level before the NTD) or additional subjects within any dose cohort may be required to determine the MTD more precisely.

In Part B, subjects may start CC-223 at the MTD and/or a lower dose level based on safety, PK and PD data from Part A. Approximately 230 subjects will be treated and evaluated for safety and preliminary antitumor activity after every two cycles of therapy for the first 2 years and, if considered still stable, every 3 cycles thereafter. Tumor types include NSCLC, GBM, HCC, NET of non-pancreatic origin, HRPBC, DLBCL, and MM. Up to 40 evaluable subjects will be enrolled in each tumor type.

3.2. Study Design Rationale

This is a Phase 1/2, multi-center, open-label study of CC-223, administered orally. CC-223 is a new investigational product which has a strong biological rationale for the treatment of subjects with advanced relapsed or refractory cancers, an unmet clinical need. Potential efficacy and toxicities associated with CC-223 were evaluated in preclinical studies. The safety and tolerability of CC-223 in humans, as well as the preliminary efficacy, will be evaluated in this trial. The study will be conducted in two parts: dose escalation (Part A) and dose expansion (Part B).

In Part A, a modified accelerated titration design (Simon, 1997) will be used to establish initial toxicity, as described above. Accelerated titration designs have been shown to be more efficient, limiting the number of subjects treated at sub-therapeutic doses, increasing the number of subjects treated at or near the ultimate Phase 2 dose without exposing more subjects to doses above the MTD and reducing the duration of the trial by doubling the dose until development of

EDMS Doc. Number: 20012337 - 16445273

toxicity (Simon, 1997). This study design also potentially requires fewer subjects since each cohort consists of one subject. This also minimizes intersubject variability (Simon, 1997).

A dose of 7.5 mg/day is proposed as the starting dose in this protocol. This dose is based on the recommendations in the March 2010 FDA Guidance for Industry entitled, "S9 Nonclinical Evaluation for Anticancer Pharmaceuticals," where a start dose for first administration in humans should be either one-tenth the STD10 in rodents, or one-sixth of the HNSTD if the nonrodent is the most appropriate species. The conversion factors from the July 2005 FDA Guidance for Industry entitled, "Estimating the Maximum Safe Starting Dose in Initial Clinical Trials for Therapeutics in Adult Healthy Volunteers," were used to convert the animal dose to the HED. The dose was converted to milligrams based on a 60-kg human body weight.

Species	Animal Dose	HED (mg/kg)	HED (mg)	Safety Factor	Start Dose (mg)
Rat	10 mg/kg STD10	1.6	96	10	9.6
Dog	1.5 mg/kg HNSTD	0.81	49	6	8.2

Since the dog was identified as the most sensitive species in the 28-day GLP toxicology studies, the start dose for the study would be 8.2 mg/day. Based on available dosage strengths of the IP, a dose of 7.5 mg/day of CC-223 is deemed to be an acceptable starting dose for this initial trial in patients.

Part B will consist of approximately 230 patients with selected tumor types to further assess the safety profile and provide preliminary efficacy information. Anti-tumor activity will be monitored by tumor type. For Part B, the selected dosing schedule will be based on the safety and PK-PD (biomarker) data from Part A.

The design of the study is presented in Figure 1.

EDMS Doc. Number: 20012337 - 16445273

Figure 1: Overall Study Design

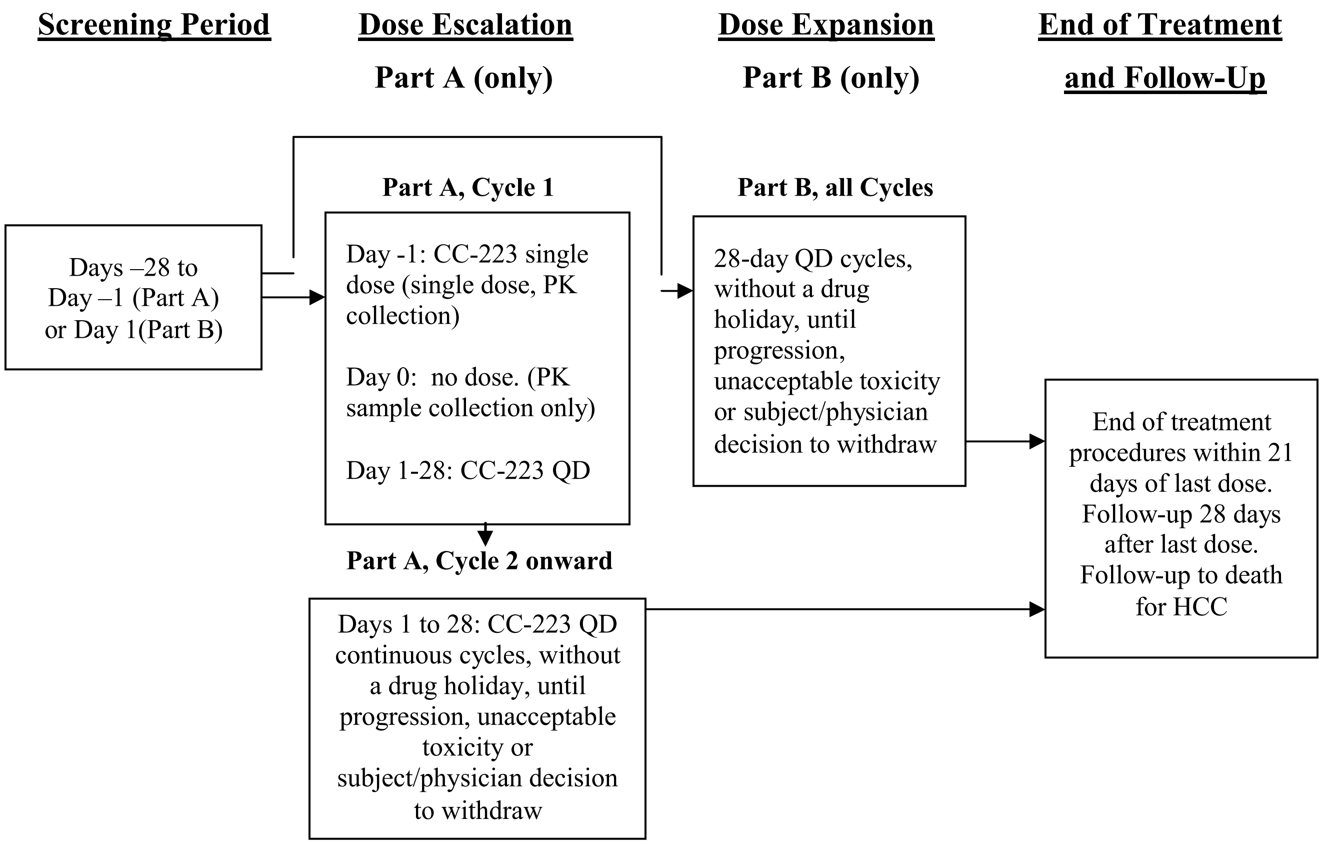

3.3. Study Duration

In Part A, in Cycle 1, each subject will be administered a single oral dose of CC-223 (Day -1), followed by a 48-hour observation and PK sample collection period. This is followed on Day 1 by daily uninterrupted dosing for 28 days (Cycle 1 =30 days). If necessary for logistical reasons, Day 1 may be delayed by up to 4 days. If subjects continue therapy with CC-223, subsequent cycles are 28 days long, with continuous dosing from Day 1 to 28 with no dosing holiday between cycles.

In Part B, subjects receive continuous dosing with 28-day cycles from the beginning—there is no initial single dose observation period. Therapy may be discontinued if there is evidence of disease progression, but subjects can continue to receive CC-223 as long as the Investigator considers they derive benefit from treatment. Therapy will be discontinued if there is unacceptable toxicity or if the subject decides to withdraw from the study.

Enrollment is expected to begin in mid-2010 and is expected to occur over approximately 36 months. Completion of active treatment and subject follow-up is expected to take up to an additional 24 months but will be extended for subjects with indolent NET, or for subjects with HCC requiring follow-up to death.

EDMS Doc. Number: 20012337 - 16445273

4. TABLE OF EVENTS

Table 2: Table of Events

Tests and Observations	Part A and B Screening[b]		Treatment Cycles 1 to X (Every 28 Days) Continuous Dosing[a]											End of Treatment	Follow-up	
			Part A: Cycle 1 Single Dose		Parts A and B: Cycle 1 Continuous Dosing[b]					Subsequent Cycles: Continuous Dosing[c]						
	Days –28 to Day –1 (Part A) or Day 1 (Part B) pre-dose[d]	Days –14 to Day–1 (Part A) or Day 1 (Part B) pre-dose[b]	Single Dose (Day -1)	Day 0	Day 1	Day 2	Day 8	Day 15	Day 22[e]	Day 1	Day 8[f]	Day 15[g]	Day 22[f]	Within 21 Days After Last Dose	28 Days After Last Dose	HCC Cohort
Written informed consent and contraceptive counseling[d]	X															
Medical and surgical history, demographics	X															
Concomitant medications and procedures	X		X	X	X		X	X	X[e]	X	X	X	X	X		
Physical examination / ECOG performance status	X		X		X					X				X		
Vital signs/weight/height[h]	X		X	X	X		X	X	X	X	X	X	X	X		
Pregnancy test[i]		X	X[j]		X					X				X		
Fasting amylase, lipase, cholesterol and triglycerides, TSH, fT4, LDH, CK, immunoglobulins and T-cell subsets (CD4+ and CD8+)[k]		X								X				X		

EDMS Doc. Number: 20012337 - 16445273

Table 2: Table of Events (Continued)

Tests and Observations	Part A and B Screening[b]		Treatment Cycles 1 to X (Every 28 Days) Continuous Dosing[a]											End of Treatment	Follow-up	
			Part A: Cycle 1 Single Dose		Parts A and B: Cycle 1 Continuous Dosing[b]					Subsequent Cycles: Continuous Dosing[c]						
	Days –28 to Day –1 (Part A) or Day 1 (Part B) pre-dose[d]	Days –14 to Day–1 (Part A) or Day 1 (Part B) pre-dose[b]	Single Dose (Day –1)	Day 0	Day 1	Day 2	Day 8	Day 15	Day 22[e]	Day 1	Day 8[e]	Day 15[g]	Day 22[f]	Within 21 Days After Last Dose	28 Days After Last Dose	HCC Cohort
HbA1c		X								X[l]				X[m]		
Uric Acid[k]		X								X				X		
CBC with differential[n]		X	X		X		X	X	X	X	X	X	X	X		
Fasting chemistry[o]		X	X		X		X	X	X	X	X	X	X	X		
HCC lab panel[p]		X			X					X				X		
Fasting insulin, c-peptide and glucose		X					X[q]							X		
Blood glucose home monitoring (fingerstick)[r]				X	X		X	X	X	X	X	X	X	X		
Urinalysis[s, m]	X		X		X		X	X	X	X	X	X	X	X		
Urine Collection[t]			X	X	X											
PT/INR/PTT (Only for subjects on warfarin)[u]		X	X		X		X	X	X	X	X	X	X	X		
12-lead ECG (Triplicate)[v, m]	X		X		X			X		X						
LVEF (MUGA or ECHO)	X									X[w]				X[x]		

EDMS Doc. Number: 20012337 - 16445273

Table 2: Table of Events (Continued)

Tests and Observations	Part A and B Screening[b]		Treatment Cycles 1 to X (Every 28 Days) Continuous Dosing[a]											End of Treatment	Follow-up	
			Part A: Cycle 1 Single Dose		Parts A and B: Cycle 1 Continuous Dosing[b]					Subsequent Cycles: Continuous Dosing[c]						
	Days –28 to Day –1 (Part A) or Day 1 (Part B) pre-dose[d]	Days –14 to Day–1 (Part A) or Day 1 (Part B) pre-dose[b]	Single Dose (Day –1)	Day 0	Day 1	Day 2	Day 8	Day 15	Day 22[e]	Day 1	Day 8[e]	Day 15[g]	Day 22[f]	Within 21 Days After Last Dose	28 Days After Last Dose	HCC Cohort
Tumor assessments: Brain, chest, abdomen and pelvis CT/MRI for solid tumors and NHL/DLBCL[y]	X									X				X		
Tumor assessments[z]: Bone marrow evaluation for MM and NHL/DLBCL	X							X		X[z]				X		
MM serum/urine panel[aa]		X								X				X		
PET scan (Part B Only)[bb]	X							X		X						
Pharmacogenomic sampling[cc]	X															
Pharmacokinetic sampling[dd]			X	X	X	X		X								
PD Biomarker sampling [ee](Part A Only)		X	X		X		X	X	X	X[ff]						
PD Biomarker sampling[ee] (Part B Only)	X				X			X								

EDMS Doc. Number: 20012337 - 16445273

Table 2: Table of Events (Continued)

Tests and Observations	Part A and B Screening[b]		Treatment Cycles 1 to X (Every 28 Days) Continuous Dosing[a]											End of Treatment	Follow-up	
			Part A: Cycle 1 Single Dose		Parts A and B: Cycle 1 Continuous Dosing[b]					Subsequent Cycles: Continuous Dosing[c]						
	Days –28 to Day –1 (Part A) or Day 1 (Part B) pre-dose[d]	Days –14 to Day–1 (Part A) or Day 1 (Part B) pre-dose[b]	Single Dose (Day –1)	Day 0	Day 1	Day 2	Day 8	Day 15	Day 22[e]	Day 1	Day 8[e]	Day 15[g]	Day 22[f]	Within 21 Days After Last Dose	28 Days After Last Dose	HCC Cohort
CC-223 administration[gg]			X		X	X	X	X	X	X	X	X	X			
AE Monitoring[hh]	X		X	X	X		X	X	X	X	X	X	X	X	X	
Archival Tumor[ii]	X															
Tumor Biopsy[jj]	X							X						X		
Salvage resection & biopsy with post op MRI (GBM only)								X[kk]								
NET-specific evaluations[ll]		X			X					X				X		
Survival status[mm]																X

[a] All visits from Cycle 1 Day 8 onwards allow a ±1 day window.
[b] Screening assessments completed within 72 hours of Part A Day -1 or Part B Day 1 do not need to be repeated on Day 1.
[c] After 2 years of treatment, clinic visits may be reduced to every 3 cycles. In such cases, subjects between clinic visits will be contacted by phone every 6-weeks for routine safety assessment, or be assessed in-clinic.
[d] Written informed consent and screening procedures will be performed within 28 days of Day –1 (Part A) or Day 1 (Part B), except for laboratory tests, which must be performed within 14 days of Day –1 (Part A) or Day 1 (Part B). Double contraception must be practiced from the time consent is obtained.
[e] For GBM only. With the exception of AE reporting, most C1D22 procedures may be omitted during the post-resection recovery period when subjects are not taking CC-223. During that time, supportive postoperative concomitant medication/procedures that are unrelated to study medication need not be documented on the eCRF.
[f] Cycle 2 onwards: Day 8 and Day 22 visits may be omitted at discretion of the Investigator in the absence of ≥ grade 2 CC-223 related toxicity.
[g] Cycle 3 onwards: Day 15 visit may be omitted at discretion of the Investigator in the absence of ≥ grade 2 CC-223 related toxicity.
[h] Height and weight recorded at Screening. Thereafter, weight is recorded on Cycle 1, Day –1 (Part A) and Day 1 of each cycle (Parts A and B) and End of Treatment (Parts A and B).
[i] Serum or urine pregnancy test to be obtained in females of childbearing potential within 72 hours prior to Day 1 dosing for each cycle. After 2 years of treatment, pregnancy testing will be conducted no more frequently than monthly when subjects attend clinic.
[j] For Part A patients, pregnancy test must be obtained within 48 hours prior to Cycle 1, Day –1 dosing. If negative, it does not need repeating within 72 hours prior to Cycle 1, Day 1.

EDMS Doc. Number: 20012337 - 16445273

[k] After 2 years of treatment, TSH, fT4, LDH, CK, immunoglobulins and T-cells, uric acid, and urinalysis may be omitted.

[l] HbA1c to be performed on Day 1 of odd-numbered cycles only, starting in Cycle 3. After 2 years of treatment, performed every 3 cycles and EOT.

[m] Omitted if last performed within the prior 28 days.

[n] CBC, drawn predose, includes: hemoglobin, hematocrit, red blood cell count with indices (mean corpuscular volume, mean corpuscular hemoglobin and mean corpuscular hemoglobin concentration), white blood cell count with absolute differential (neutrophils, lymphocytes, monocytes, eosinophils and basophils) and platelet count.

[o] Fasting serum chemistry, drawn predose, includes; albumin, total protein, bicarbonate, calcium, phosphorus, chloride, serum creatinine, serum urea/BUN, glucose, c-peptide, potassium, sodium, chloride, total bilirubin, AST, ALT and alkaline phosphatase.

[p] For HCC subjects only. Alphafetoprotein (AFP) at Screening, Day 1 of every cycle, and End of Treatment. HBV and HCV markers (HBsAg, anti-HBS, anti-Hbc, anti-HCV, HBV viral load) at Screening only. For HBsAg positive subjects, HBV viral load at Screening, on Day 1 of every cycle, and End of Treatment.

[q] On Cycle 1, Day 8, fasting insulin, c-peptide and blood glucose will be drawn at predose (< 15 mins prior to dosing), 1.5hr and 3hr after dosing. The predose c-peptide/glucose sample may be part of the routine chemistry panel.

[r] Frequency of testing will be determined by Investigator, based on subject's most recent blood glucose status. At minimum, glucose home monitoring will be documented daily (fasting, morning) in Cycle 1 and the results reviewed each clinic visit. At the discretion of the Investigator, home monitoring may be eliminated in Cycle 2 onwards in the absence of any fasting grade 2 hyperglycemia throughout Cycle 1 (see Appendix C. Section 18.3.1).

[s] Dipstick, with microscopy in event of positive (1+ or greater) blood or protein and 24-hour collection for creatinine clearance and protein quantification in the event of 2+ or greater protein.

[t] Part A: Total urine collections for PK analysis on Day -1 through Day 0 will be taken over the following times: Predose (<30 minutes prior to dosing, to completely empty the bladder), 0-4 hr and 4-8hr (while the subject is in the clinic), and 8-24hr (while the subject is at home).
Part B: Only selected US investigational sites will make total urine collections for PK analysis on Day 1 through 2 over the following times: Predose (<30 minutes prior to dosing, to completely empty the bladder), 0-4 hr and 4-8hr (while the subject is in the clinic), and 8-24hr (while the subject is at home). Refer to the Laboratory Manual for urine collection instructions.

[u] Coagulation tests are only for subjects on warfarin, performed weekly or as clinically indicated.

[v] ECGs. Part A: A triplicate 12-lead ECG (3 recordings within 2±1 minute intervals) will be performed at Screening and at the following time points during Cycle 1: predose (≤ 90 mins prior to dosing), and again between 1.5 and 3 hrs after dosing on Day -1 (Part A only), Day 1, Day 8, Day 15 and Day 22. During subsequent cycles, triplicate 12-lead ECGs will be performed on Day 1, 8, 15 and 22 predose (≤ 90 mins prior to dosing), and again between 1.5 and 3 hrs after dosing. Final ECG is at End of Treatment.
Part B: (US sites only): A triplicate 12-lead ECG (3 recordings within 2±1 minute intervals) will be performed at Screening, predose (≤ 90 mins prior to dosing), and between 1.5 and 3 hrs after dosing on Day 1 and Day 15. During Cycles 2, 3 and 4, single 12-lead ECGs will be performed on Day 1 between 1.5 and 3 hrs after dosing. Later in the study and other than at EOT, performed only if indicated and using an institutional machine with central evaluation no longer required, no ECGs are required beyond Cycle 4.
Part B (EU sites): After Screening, ECGs should be performed only as clinically indicated.
ECGs will be recorded after the subject has rested supine for at least 5 minutes. When they coincide with a blood collection or a meal, ECGs will take precedence. ECG machines, provided by a central ECG laboratory, will record at 25 mm/s and report rhythm, ventricular rate, PR-interval, QRS, QT, and QTc intervals. ECG output will be frequently uploaded to the ECG laboratory for definitive analysis and interpretation. Later in the study, ECGs may be performed using an institutional machine and will not be evaluated centrally. The SRC may reduce the requirements for ECG monitoring in Part B sites after 30 - 60 subjects are evaluated and if no clinically significant changes have been observed.

[w] Part A: Ejection fraction to be performed on Day 1 (± 7 days) of every third cycle, starting Cycle 3.
Part B: Ejection fraction to be performed on Day 1 (± 7 days) of every third cycle, starting Cycle 3 but monitoring will be discontinued after 3 post treatment studies for individual subjects if there are no clinically significant changes (ie, LVEF > 45% and < 20% change from baseline). The SRC may eliminate the requirement for LVEF monitoring in Part B after 30 - 60 subjects are evaluated if no clinically significant changes have been observed.

[x] Part A: May be omitted if last performed within the prior 56 days.
Part B: Only for subjects with significant changes from Screening. May be omitted if last performed within the prior 56 days.

[y] Tumor assessments, including imaging (CT or MRI) of the chest, abdomen and pelvis at baseline for tumors except MM and GBM. Subjects with brain lesions will have MRI brain scans at Screening and at each tumor assessment. After Screening, tumor assessments will be performed on completion of C2, C4 and C6 (ie, C3, 5 and 7/Day1±7 days) using the same scanning modalities used at baseline; after cycle 6, the frequency of tumor assessments will be every 3 months. Subjects with GBM will have a new baseline

EDMS Doc. Number: 20012337 - 16445273

MRI scan performed in Cycle 1 ≤7 days after restarting CC-223 post resection. Corticosteroids (if used) dosage must be stable ≥ 5 days prior to all scans. EOT scan need not be repeated if prior scan was < 28 days.

[z] For MM, and only for NHL/DLBCL subjects with known or suspected marrow involvement: Bone marrow aspiration and biopsy (with cytogenetic and PD biomarker analysis) at Screening, Cycle 1 Day 15±7 days, on completion of cycles 4, 8, 12 and 16 (ie, C5, 9, 13 and 17/Day1±7 days) and at End of Treatment. Cytogenetics need not be repeated if negative at Screening.

[aa] For MM subjects: Within 7 days prior to Day 1, Day 1 of subsequent cycles and at End of Treatment: Serum protein electrophoresis (SPEP) with immunoelectrophoresis (IEP) and 24 hour urine for urine protein electrophoresis (UPEP) and IEP (not repeated if negative at Screen), quantitative immunoglobulins (IgG, IgM, IgA), serum free kappa and lambda light chains and ratio, and beta-2-microglobulin.

[bb] Part B only (all tumors except MM): PET imaging at Screening, on Cycle 1, Day 15±7 and Cycle 3, Day 1±7 (not to be repeated in later cycles). The Day 15 assessment will be delayed until CC-223 has been administered continuously for at least 7 days when study drug was interrupted prior to that time. For GBM subjects, PET imaging is performed prior to salvage resection.

[cc] Pharmacogenomic sampling: Approximately 11 mL blood will be collected during Screening from all subjects (Parts A and B). Refer to the Laboratory Manual for sample collection instructions.

[dd] Pharmacokinetic sampling.

Part A only: 1 mL whole blood will be drawn for PK at the following times in Cycle 1. On Day -1: 0hr (< 15 min prior to dosing), 0.5hr, 1hr, 1.5hr, 3hr, 5hr, 8hr, 24hr and 48hr after the first dose of CC-223; Day 15: 0hr (< 15 min prior to dosing), 0.5hr, 1hr, 1.5hr, 3hr, 5hr, and 8hr after the Day 15 dose.

Part B only: selected (Part A) investigational sites: 1 mL whole blood will be drawn for PK at the following times in Cycle 1. On Day 1: 0hr (< 15 min prior to dosing), 0.5hr ±5min, 1hr ±5min, 1.5hr ±10min, 3hr ±10min, 5hr ±15min, 8hr ±15min, and 24 ±1 hr after the first dose of CC-223; Day 15: 0hr (< 15 min prior to dosing) and 1.5hr ±10min after dosing.

Part B only; HCC subjects only: 1 mL whole blood will be drawn for PK at the following times in Cycle 1. On Day 1: 0hr (< 15 min prior to dosing), 0.5hr ±5min, 1hr ±5min, 1.5hr ±10min, 3hr ±10min, 5hr ±15min, 8hr ±15min, and 24 ±1 hr after the first dose of CC-223; Day 15: 0hr (< 15 min prior to dosing) 0.5hr ±5min, 1hr ±5min, 1.5hr ±10min, 3hr ±10min, 5hr ±15min, and 8hr ±15min post dose.

All other sites/cohorts: 1 mL whole blood will be drawn in Cycle 1 predose (< 15 min prior to CC-223) and 1.5 hr ±10min post dose on Day 1 and Day 15.

Note: The Day 15 assessments at all sites will be delayed until CC-223 has been administered continuously for at least 7 days when study drug was interrupted prior to that time. If the dose of CC-223 is reduced but with (optional) subject consent, blood will be withdrawn for PK assessments predose (< 15 mins prior to dosing), 1 hour ± 10 mins, 1.5 hours ± 10 mins, 3 hours ± 10 mins, and 5 hours ± 15 mins post dose 7 – 14 days following dose reduction in order to evaluate intrasubject study drug PK. 1 mL whole blood will be drawn for PK as close as possible to the time of post-treatment tumor biopsy procedures (Day 15±7), approximately 3±1 hr after dosing with CC-223.

[ee] Pharmacodynamic Biomarker sampling.

Part A: Approximately 14 mL whole blood will be drawn for PD biomarker measurements at the following times. During Screening: No less than 1 day prior to Day -1. In Cycle 1: 0 hr (< 15 min prior to dosing) on Days -1, 1, 8, 15 and 22. In Cycle 2: Predose on Day 1 only. Approximately 12 mL whole blood will be drawn at the following times post-dose in Cycle 1. On Day -1: 1.5 hr, 3 hr and 5 hr. On Days 1, 8, 15 and 22: at 1.5 hr. In cycle 2, at 1.5 hr on Day 1 only.

Part B: US investigational sites only. A total of approximately 16 mL whole blood will be drawn for PD biomarker measurements in Cycle 1, Days 1 and 15 predose 0 hr (< 15 min prior to dosing) and 1.5 hr ±10min after dosing (when this coincides with the day of on-treatment biopsy, the 1.5 hr draw will be delayed and synchronized with the biopsy procedure). The Day 15 assessments will be delayed until CC-223 has been administered continuously for at least 7 days when study drug was interrupted prior to that time. In addition, a total of approximately 8 mL whole blood will be drawn for PD biomarkers during Screening (at the time of tumor biopsy, if applicable) and at the time of on-treatment biopsies or salvage tumor resection (Day 15±7), 3±1 hr after dosing with CC-223.

[ff] Only on Day 1 Cycle 2, not subsequent cycles.

[gg] Subjects must delay morning dosing on certain clinic days until appropriate predose blood draws (eg, PK, PD) are complete. On Day 8, food may not be consumed until 3 hrs after dosing (ie, after the 3 hr fasting insulin blood draw).

[hh] Subject diary cards will be reviewed during each clinic attendance for AE assessment. Carcinoid/NET-specific symptoms will be documented or transcribed onto a dedicated symptom eCRF. Follow-up of adverse event (AE) / serious adverse event (SAE) resolution will continue for 28 days after last dose.

[ii] Optional for Part A, mandatory for Part B. Archival tumor, as FFPE blocks or mounted sections, retrieved in all tumor types (except MM) unless single-case exemption is granted by Celgene. NET tumor tissue retrieval is optional in Part B.

[jj] Optional for Part A, mandatory for Part B (all tumors except NSCLC and NET, where it is optional).

Either core needle or excisional tumor biopsies from one or more sites, or aspiration of bone marrow (with tumor involvement), during Screening and while on treatment (Cycle

EDMS Doc. Number: 20012337 - 16445273

1, between Day 15±7 days) for analysis of genetic mutations and biomarkers of CC-223 activity. The Day 15 biopsy will be delayed until CC-223 has been administered continuously for at least 7 days when study drug was interrupted prior to that time. For GBM, a tumor biopsy at Screening will not be required—archival tumor will be used for pre-treatment biomarker evaluation. On-treatment, a minimum of 300 mg of tumor will be obtained during salvage tumor resection on Day 15±7. See Laboratory Manual for sample collection instructions. CC-223 dose should be administered 3±1 hrs prior to all tumor biopsy procedures. Approximately 8 mL whole blood drawn for PD biomarkers (US sites only), and 1 mL whole blood drawn for PK (all sites). Additional optional tumor biopsy procedures may be obtained if appropriate and with subject consent during later treatment cycles or after treatment is discontinued.

[kk] The timing of procedures scheduled for Day 8, 15 and 22 will be adjusted to accommodate the timing of surgery. Mandatory salvage resection on Day 15±7 must yield minimum 300 mg fresh frozen tumor for PD biomarkers and drug assay. See Laboratory Manual for details. CC-223 is administered 3±1 hrs prior to resection. Approximately 12 mL whole blood drawn for PD biomarkers, and 1 mL whole blood drawn for PK, at time of resection. Brain MRI < 7 days prior to restarting CC-223 post operatively.

[ll] NET-specific evaluations as follows:

i) Fasting Chromogranin A, and any other hormones known to be secreted by the tumor (chromogranin B, gastrin, glucagon, serotonin etc.): Screening and Day 1±7 of odd - numbered cycles starting with Cycle 3 (3, 5, 7 etc.) and EOT. After 2 years of treatment, after every 3 cycles. If normal at Screening, these do not need repeating at subsequent time points.

ii) Carcinoid/NET-specific symptom questionnaire in Screening, on Day 1 of each cycle for 2 years, then on Day 1 of every 3 cycles, and EOT.

[mm] HCC cohort will be followed every 2 months (± 1 week) for survival status until death, or until lost to follow-up/withdrawal of consent.

EDMS Doc. Number: 20012337 - 16445273

5. PROCEDURES

The study procedures to be conducted for each subject enrolled in the study are outlined in Table 2 and described in the text that follows.

5.1. Screening (Parts A and B)

Screening laboratory procedures must be performed within 5 days of Day -1 (Part A) or 14 days of Day 1 (Part B). However, screening assessments completed within 72 hours prior to Day -1/Day 1 do not need to be repeated on Day 1. Study assessments may be done up to 72 hours prior to Day 1 for all other cycles.

Written informed consent and screening procedures must be performed within 28 days of Day –1 (Part A) or 28 days of Day 1 (Part B):

- Documentation of written informed consent and agreement to begin double contraceptive practice from the day of consent
- Confirmation of inclusion/exclusion criteria
- Demographic information, including date of birth, race and gender
- Complete medical and surgical history
- Recording of prior concomitant medications and procedures (include all medications taken and procedures performed within 28 days prior to study consent)
- Weight and height
- Vital signs (blood pressure, pulse rate, respiratory rate, and temperature)
- Complete physical examination
- Eastern Cooperative Oncology Group Performance Status (ECOG PS)
- 12-lead triplicate ECG
- LVEF by ECHO or MUGA
- PD Biomarker sample (Part B, US sites only and synchronized with tumor biopsy where applicable [GBM patients do not have a Screening biopsy but should have a PD Biomarker sample drawn].)
- Tumor assessments (for all tumors except MM): Imaging studies of chest, abdomen and pelvis (including other sites, if appropriate) except for GBM. Brain scan for subjects with known brain lesions. For subjects with GBM undergoing salvage tumor resection during Cycle 1 of study treatment, a new baseline brain MRI should be obtained post-operatively within 7 days prior to restarting CC-223 and when the subject has been on a stable dose of glucocorticoids for at least 5 days.
- Tumor assessments (for MM, and only NHL/DLBCL subjects with known or suspected marrow involvement): Bone marrow aspiration and biopsy (with cytogenetic and PD biomarker analysis).

EDMS Doc. Number: 20012337 - 16445273

- Urinalysis: Dipstick, with microscopy in the event of positive (1+ or greater) blood or protein and 24-hour collection for creatinine clearance and protein quantification in the event of 2+ or greater protein.
- PET Scan (Part B only)
- Recording of adverse events
- Recording of carcinoid/NET-specific symptoms (Part B NET cohort)
- Pharmacogenomic blood sampling
- Tumor biopsy (Part A optional, Part B mandatory (all tumors except NSCLC and NET, where it is optional): Excision or core needle biopsy from one or more sites, or aspiration of bone marrow (with tumor involvement) no sooner than 2 weeks after discontinuing prior anticancer therapy. Bronchoscopic biopsy of central squamous cell lung carcinoma is not permitted. Screening tumor biopsy is not required in subjects with GBM.
- Archival Tumor (Part A optional, Part B mandatory): All subjects, except those with MM or NET, will have archival tumor (formalin-fixed paraffin embedded [FFPE] block or sections) retrieved for biomarker and/or gene mutation analysis. NET archival tumor is optional. In the absence of an optional Screening biopsy specimen from NSCLC subjects, the adequacy of archival tissue for gene mutation must be confirmed before starting CC-223.

The following laboratory assessments will be performed within 5 days of Day –1 (Part A) or 14 days of Day 1 (Part B):

- Insulin (fasting)
- Hematology: Complete blood count (CBC) with differential and platelet counts.
- Fasting serum chemistries: Na, K, Cl, HCO_3, Ca, glucose, c-peptide, BUN, creatinine, total bilirubin, AST, ALT, alkaline phosphatase, albumin, phosphorus, total protein
- Pregnancy test (serum or urine) in females of childbearing potential within 72 hours of first dose. An alternative to hCG testing may be used to rule out pregnancy in cases of hCG-secreting NET.
- Amylase, lipase, cholesterol and triglycerides, TSH, fT4, LDH, creatine kinase (CK), immunoglobulins and T-cell subsets (CD4+ and CD8+)
- HbA1c
- Uric Acid
- PT/INR/PTT, only for subjects on warfarin
- Part A Only: PD Biomarker sampling (no sooner than 1 day prior to Day -1)
- For MM subjects only: Serum protein electrophoresis (SPEP) with immunoelectrophoresis (IEP), 24 hour urine for urine protein electrophoresis (UPEP) and IEP, quantitative immunoglobulins (IgG, IgM, IgA), serum free kappa and lambda light chains and ratio, and beta-2-microglobulin.

EDMS Doc. Number: 20012337 - 16445273

- For HCC subjects only: AFP, HBsAg, anti-HBS, anti-HBc, anti-HCV, HBV viral load.
- For NET subjects only: Fasting blood chromogranin A, with other hormones (eg, chromogranin B, gastrin, glucagon, serotonin) known to be secreted by the tumor.

Note: In the event subjects fail Screening, minimal information will still be documented on the electronic case report forms (eCRFs), per database instructions.

5.2. During Treatment

Cycle 1, Day -1 (Part A only: single dose administration)

The following assessments will be performed on the first day of initial treatment with CC-223. Baseline (Day 1) laboratory assessments do not need to be repeated if screening laboratory findings were obtained within 72 hours of start of initial dosing with CC-223.

- Physical examination, ECOG PS
- Vital signs, weight
- Recording of adverse events
- Recording of concomitant medications and procedures
- Hematology: CBC with differential and platelet counts.
- Fasting serum chemistries: Na, K, Cl, HCO_3, Ca, glucose, c-peptide, BUN, creatinine, total bilirubin, AST, ALT, alkaline phosphatase, albumin, phosphorus, total protein
- Urinalysis: Dipstick, with microscopy in the event of positive blood or protein
- Urine collection in clinic (predose (< 30 min prior to dosing), 0-4 h and 4-8 hr intervals); Urine collection at home (8-24 hrs)
- Serum or urine pregnancy test in females of childbearing potential within 72 hours of Cycle 1, Day -1
- Pharmacokinetic sampling: 0 hr (pre-dose, < 15 min prior to dosing), 0.5, 1, 1.5, 3, 5, 8 hr post dose
- PD Biomarker sampling: 0 hr (pre-dose, < 15 min prior to dosing) and 1.5, 3, and 5 hr post dose
- 12-lead triplicate ECG predose (≤ 90 min prior to dosing), between 1.5 and 3 hours after dosing
- PT/INR/PTT, only for subjects on warfarin
- Blood glucose home monitoring: Subjects taught how to use a glucometer and document results of daily self-testing

Cycle 1, Day 0 Assessments (Part A Only)

- Vital signs
- Pharmacokinetic sampling (24 hr post dose)

EDMS Doc. Number: 20012337 - 16445273

- Urine collection at home (8-24 hrs)
- Recording of adverse events
- Recording of concomitant medications and procedures
- Blood glucose fingerstick testing

Cycle 1, Day 1 Assessments

For Part A patients, the following laboratory tests do not need repeating on Day 1, as long as they were performed within 4 days prior Day 1: CBC, fasting chemistry, urinalysis and PT/INR/PTT for patients on warfarin.

- Vital signs, weight
- Physical examination, ECOG PS
- Recording of adverse events
- Recording of carcinoid/NET-specific symptoms (Part B NET cohort)
- Recording of concomitant medications and procedures
- Part B Only: Serum or urine pregnancy test in females of childbearing potential performed within 72 hours of first dose. An alternative to hCG testing may be used to rule out pregnancy in cases of hCG-secreting NET.
- Hematology: CBC with differential and platelet counts.
- Fasting serum chemistries: Na, K, Cl, HCO_3, Ca, glucose, c-peptide, BUN, creatinine, total bilirubin, AST, ALT, alkaline phosphatase, albumin, phosphorus, total protein
- Blood glucose fingerstick testing
- Urinalysis: Dipstick, with microscopy in the event of positive (1+ or greater) blood or protein and 24-hour collection for creatinine clearance and protein quantification in the event of 2+ or greater protein
- Part B Only: At selected (US Part A) sites - urine collection in clinic (predose < 30 min prior to dosing), 0-4 h and 4-8 hr intervals); Urine collection at home (8-24 hrs)
- PT/INR/PTT, only for subjects on warfarin
- US sites only: 12-lead triplicate ECG predose (≤ 90 min prior to dosing), between 1.5 and 3 hours after dosing
- Pharmacokinetic sampling.
  - Part A: 48 hr post Day -1 dose blood draw.
  - Part B:
    - Selected (US Part A and HCC subjects) sites. 0 hr (predose, < 15 min prior to dosing), 0.5 hr ±5 min, 1 hr ±5 min, 1.5 hr ±10 min, 3 hr ±10 min, 5 and 8 hrs ±15 mins post dose

EDMS Doc. Number: 20012337 - 16445273

    - All other sites in US and EU. Predose (< 15 min prior to dosing) and 1.5 hr ±10 min post dose.
- PD Biomarker sampling (US sites only): 0 hr (pre-dose, < 15 min prior to dosing) and 1.5 hrs ±10 min post dose.
- For HCC subjects only: AFP. HBV viral load for HBsAg positive subjects.

Cycle 1, Day 2 (Part B Only)

- Pharmacokinetic sampling (at selected US Part A sites and HCC subjects only). Predose (< 15 min prior to CC-223, ie, approximately 24 hours after the Day 1 dose).

Cycle 1, Day 8, 15 and 22 (all ±1 day)

- Vital signs
- Hematology: CBC with differential and platelet counts.
- Fasting serum chemistries: Na, K, Cl, HCO_3, Ca, glucose, c-peptide, BUN, creatinine, total bilirubin, AST, ALT, alkaline phosphatase, albumin, phosphorus, total protein
- Urinalysis: Dipstick, with microscopy in the event of positive (1+ or greater) blood or protein and 24-hour collection for creatinine clearance and protein quantification in the event of 2+ or greater protein
- Blood glucose fingerstick testing
- PT/INR/PTT, only for subjects on warfarin
- Fasting insulin, c-peptide and blood glucose only on Day 8, 0 hr (predose, < 15 min prior to dosing), 1.5 and 3 hrs post dose
- Pharmacokinetic sampling.
  - Part A on Day 15 only: 0 hr (pre-dose, < 15 min prior to dosing), 0.5, 1, 1.5, 3, 5, and 8 hr after dosing
  - Part B:
    - All subjects except HCC subjects. 0 hr (pre-dose, < 15 min prior to dosing) and 1.5 hr ± 10 min post dose on Day 15 only. Sampling will be delayed until CC-223 has been administered continuously for at least 7 days when study drug was interrupted prior to that time.
    - HCC subjects. 0hr (< 15 min prior to dosing) 0.5hr ±5min, 1hr ±5min, 1.5hr ±10min, 3hr ±10min, 5hr ±15min, and 8hr ±15min post dose. Sampling will be delayed until CC-223 has been administered continuously for at least 7 days when study drug was interrupted prior to that time.
    - As close as possible to the time of any on-treatment tumor biopsy procedure

EDMS Doc. Number: 20012337 - 16445273

- PD biomarker sampling.
  - Part A on Day 8, 15 and 22: 0 (pre-dose, < 15 min prior to dosing) and 1.5 hr (post dose).
  - Part B on Day 15 (US sites only): 0 (pre-dose, < 15 min prior to dosing) and 1.5 hr ± 10min (post dose). Both blood draws will be taken while the subject is fasting. Sampling will be delayed until CC-223 has been administered continuously for at least 7 days when study drug was interrupted prior to that time. If tumor biopsy is performed on the same day as the Day 15 PD blood draw, the 1.5 hr post dose sample will be delayed and synchronized with the biopsy (see below)
- ECGs.
  - Part A: 12-lead triplicate ECG (3 recordings within 2 ±1 minute intervals) predose (≤90 mins prior to dosing), between 1.5 and 3 hours after dosing
  - Part B: US sites only record 12-lead triplicate ECGs predose, between 1.5 and 3 hours after dosing only on Day 15. EU sites record 12-lead single ECGs only if clinically indicated.
- Tumor biopsy on Day 15 (± 7 days) (optional in Part A; mandatory in Part B for all tumors except NSCLC and NET, where it is optional) from the same site biopsied in Screening, if possible. Bronchoscopic biopsy of central squamous cell lung carcinoma is not permitted. Biopsy will be delayed until CC-223 has been administered continuously for at least 7 days when study drug was interrupted prior to that time. For subjects with GBM, salvage resection will be performed with appropriate tissue saved for biopsy specimens. For subjects with MM, bone marrow aspiration will be performed. The following additional procedures will be performed in conjunction with the tumor biopsy procedure (see laboratory manual for details):
  - (i) CC-223 administered 3±1 hr prior to tumor biopsy
  - (ii) Minimum 200 mg tumor tissue prepared for PD biomarker assay (300 mg for GBM tumor PD/PK)
  - (iii) Whole blood drawn for PD (US sites only) and PK (all sites) as close as possible to the same time as the tumor biopsy
- For GBM subjects only: Brain MRI scan postoperatively, within 7 days of restarting CC-223
- Part B: PET scan on Day 15±7 days (as close as possible to the tumor biopsy procedure) for all but MM subjects. PET scan will be delayed until CC-223 has been administered continuously for at least 7 days when study drug was interrupted prior to that time.
- Review of diary card, recording of appropriate adverse events and daily blood glucose home monitoring results
- Recording of concomitant medications and procedures

EDMS Doc. Number: 20012337 - 16445273

Note: For GBM subjects only, but with the exception of AE reporting, C1D22 procedures may be omitted during the post-resection recovery period while subjects are not taking CC-223. During that time, postoperative concomitant medications/procedures exclusively for surgical support and unrelated to study medication need not be documented on the eCRF.

Subsequent Cycles for up to 2 Years, Day 1 (± 1 day)

- Physical examination, ECOG PS
- Vital signs, weight
- Recording of adverse events and daily home blood glucose monitoring results
- Recording of carcinoid/NET-specific symptoms (Part B NET cohort)
- Recording of concomitant medications and procedures
- Hematology: CBC with differential and platelet counts
- Fasting serum chemistries: Na, K, Cl, HCO_3, Ca, glucose, c-peptide, BUN, creatinine, total bilirubin, AST, ALT, alkaline phosphatase, albumin, phosphorus, total protein
- Blood glucose fingerstick testing
- PT/INR/PTT, only for subjects on warfarin
- Amylase, lipase, cholesterol and triglycerides, TSH, fT4, LDH, CK, immunoglobulins and T-cell subsets (CD4+ and CD8+)
- Uric acid
- Urinalysis: Dipstick, with microscopy in the event of positive (1+ or greater) blood or protein and 24-hour collection for creatinine clearance and protein quantification in the event of 2+ or greater protein
- Serum or urine pregnancy test in females of childbearing potential within 72 hours of Day 1 of each cycle. An alternative to hCG testing may be used to rule out pregnancy in cases of hCG-secreting NET
- ECGs
  - Part A: 12-lead triplicate ECG predose and between 1.5 and 3 hrs after dosing
  - Part B: (US sites only) 12-lead single ECG between 1.5 and 3 hrs after dosing for Cycle 2, 3 and 4 only, and as clinically indicated. EU sites perform 12-lead single ECG only as clinically indicated
- For MM subjects only: SPEP with IEP, 24 hour urine for UPEP with IEP (only if positive at Screen), quantitative immunoglobulins (IgG, IgM, IgA), serum free kappa and lambda light chains and ratio, and beta-2-microglobulin
- Part A subjects, Cycle 2 only: PD biomarker sampling Day 1, 0 hr (pre-dose, <15 min prior to dosing) and 1.5 hr (post dose). Blood samples will be collected while the subject is fasting
- Odd-numbered cycles only (starting Day 1, Cycle 3): HbA1c

EDMS Doc. Number: 20012337 - 16445273

- Every third cycle (starting Day 1 ± 7 days, Cycle 3): LVEF by same modality used during screening. In Part B, LVEF assessment may be discontinued after Cycle 9 if there are no clinically significant changes from Screening
- For HCC subjects only: AFP. HBV viral load for HBsAg positive subjects
- For NET subjects only: Fasting blood hormones on Day 1±7 if found to be abnormal. at Screening, every odd-numbered cycle starting with Cycle 3 (C3, 5, 7, etc.)
- Part B: PET scan on only Cycle 3, Day 1 concurrent with the first restaging evaluation. Combined PET/CT imaging is acceptable
- Tumor assessments (for all tumors except MM): repeated on completion of Cycle 2, 4 and 6 (ie, Cycle 3, 5 and 7/Day 1±7 days), using the same scanning modalities used during Screening. After cycle 6, the frequency of tumor assessments will be every 3 months (eg, Cycle 10 and 13/Day1±7 days, etc.)
- Tumor assessment (for MM, and only NHL/DLBCL subjects with known or suspected marrow involvement): Bone marrow aspiration and biopsy (with PD biomarker analysis, and cytogenetic analysis if abnormality present at Screening) repeated between on completion of Cycle 4, 8, 12 and 16 only (ie, Cycle 5, 9 13 and 17/Day 1±7 days). Cytogenetics need not be repeated if normal at Screening

Subsequent Cycles for up to 2 Years, Day 8, 15 and 22 (all ± 1 day)

- Vital signs
- Hematology: CBC with differential and platelet counts
- Fasting serum chemistries: Na, K, Cl, HCO_3, Ca, glucose, c-peptide, BUN, creatinine, total bilirubin, AST, ALT, alkaline phosphatase, albumin, phosphorus, total protein
- Urinalysis: Dipstick, with microscopy in the event of positive (1+ or greater) blood or protein and 24-hour collection for creatinine clearance and protein quantification in the event of 2+ or greater protein
- Blood glucose fingerstick testing (if required)
- PT/INR/PTT, only for subjects on warfarin
- ECG
  - Part A: 12-lead triplicate ECG predose, and between 1.5 and 3 hrs after dosing
  - Part B: 12-lead single ECG only as clinically indicated
- Additional optional tumor biopsies may be requested
- Review of diary card, recording of appropriate adverse events, daily blood glucose home monitoring results (if required), and NET-specific symptoms in the Part B NET cohort
- Recording of concomitant medications and procedures

Note: Starting during Cycle 2, the Day 8 and/or Day 22 clinic visits and assessments may be omitted at the discretion of the Investigator in the absence of any ≥ grade 2 CC-223 related

EDMS Doc. Number: 20012337 - 16445273

toxicity. From Cycle 3, the Day 15 clinic visit and routine weekly assessments may also be omitted in the absence of similar toxicity.

Every 3 Cycles After 2 Years, Day 1 (± 7 days)

After 2 years of treatment, in-clinic visits may be reduced to every 3 cycles. Between clinic visits, subjects will also be assessed every 6 weeks (± 1 week) by telephone (or by optional in-clinic visit) for routine safety assessments as indicated below.

- Physical examination, ECOG PS.
- Vital signs, weight.
- Recording of adverse events, also during between-clinic 6-week assessments.
- Recording of carcinoid/NET-specific symptoms.
- Recording of concomitant medications and procedures, also during 6-week assessments.
- Hematology: CBC with differential and platelet counts.
- Fasting serum chemistries: Na, K, Cl, HCO_3, Ca, glucose, c-peptide, BUN, creatinine, total bilirubin, AST, ALT, alkaline phosphatase, albumin, phosphorus, total protein.
- Blood glucose fingerstick testing, only when indicated because of persisting hyperglycemia.
- PT/INR/PTT, only for subjects on warfarin.
- Serum or urine pregnancy test in females of childbearing potential within 72 hours prior to Day 1 of every 3rd cycle. An alternative to hCG testing may be used to rule out pregnancy in cases of hCG-secreting NET.
- ECG. 12-lead single ECG using local machine only if clinically indicated.
- HbA1c.
- LVEF. Only if clinically indicated.
- Fasting blood hormones on Day 1±7 if found to be abnormal.
- Tumor assessments, using the same scanning modalities used during Screening.

5.3. Post-Treatment Assessments

End of Treatment Assessments for Subjects with Less Than 2 Years of Treatment

When a subject discontinues treatment, End of Treatment assessments are to be performed within 21 days after the last dose.

- Physical examination, ECOG PS, Weight
- Recording of concomitant medications and procedures
- Vital signs

EDMS Doc. Number: 20012337 - 16445273

- ECG
  - Part A: 12-lead triplicate ECG
  - Part B: 12-lead single ECG using local machine only if clinically indicated
- For MM subjects only: SPEP with IEP, 24 hour urine for UPEP with IEP (only if positive at Screen), quantitative immunoglobulins (IgG, IgM, IgA), serum free kappa and lambda light chains and ratio, and beta-2-microglobulin
- LVEF using MUGA or ECHO (by the same method used in screening). This may be omitted if performed within the prior 56 days
- Hematology: CBC with differential and platelet counts
- Fasting serum chemistries: Na, K, Cl, HCO_3, Ca, glucose, c-peptide, BUN, creatinine, total bilirubin, AST, ALT, alkaline phosphatase, albumin, phosphorus, total protein
- Blood glucose fingerstick testing (if required)
- PT/INR/PTT, only for subjects on warfarin
- Fasting insulin
- Amylase, lipase, cholesterol and triglycerides, TSH, fT4, LDH, CK, immunoglobulins and T-cell subsets (CD4+ and CD8+)
- Uric acid
- HbA1c. This may be omitted if performed within the prior 28 days
- Urinalysis: Dipstick, with microscopy in the event of positive (1+ or greater) blood or protein and 24-hour collection for creatinine clearance and protein quantification in the event of 2+ or greater protein
- Serum or urine pregnancy test in females of childbearing potential. An alternative to hCG testing may be used to rule out pregnancy in cases of hCG-secreting NET
- Tumor measurements: Imaging studies of brain, chest and abdomen and other sites are repeated, as appropriate, using the same scanning modalities used during screening
- Tumor assessment (for MM, and only NHL/DLBCL subjects with known or suspected marrow involvement): Bone marrow aspiration and biopsy (with PD biomarker and cytogenetic analysis). Cytogenetics need not be repeated if no abnormality was present at Screening)
- Additional optional tumor biopsies may be requested if readily accessible and with subject consent
- Recording of adverse events and daily blood glucose results (if appropriate)
- Recording of carcinoid/NET-specific symptoms (Part B NET cohort)
- For HCC subjects only: AFP. HBV viral load for HBsAg positive subjects
- For NET subjects only: Fasting blood hormones

EDMS Doc. Number: 20012337 - 16445273

End of Treatment Assessments for NET Subjects With More Than 2 Years of Treatment

When a subject discontinues treatment, End of Treatment assessments are to be performed within 21 days after the last dose.

- Physical examination, ECOG PS, Weight
- Recording of concomitant medications and procedures
- Recording of carcinoid/NET-specific symptoms
- Vital signs
- ECG. 12-lead single ECG using local machine, only if clinically indicated
- LVEF using MUGA or ECHO (by the same method used in screening) only if clinically indicated. This may be omitted if performed within the prior 56 days
- Hematology: CBC with differential and platelet counts
- Fasting serum chemistries: Na, K, Cl, HCO_3, Ca, glucose, c-peptide, BUN, creatinine, total bilirubin, AST, ALT, alkaline phosphatase, albumin, phosphorus, total protein
- Blood glucose fingerstick testing (if required)
- PT/INR/PTT, only for subjects on warfarin
- HbA1c. This may be omitted if performed within the prior 28 days
- Serum or urine pregnancy test in females of childbearing potential. An alternative to hCG testing may be used to rule out pregnancy in cases of hCG-secreting NET
- Tumor measurements: Imaging studies of brain, chest and abdomen and other sites are repeated, as appropriate, using the same scanning modalities used during screening
- Additional optional tumor biopsies may be requested if readily accessible and with subject consent
- Recording of adverse events, and daily blood glucose results (if appropriate)
- Fasting NET-specific blood hormones

Post-Treatment Follow-Up

Subjects will be contacted 28 days after the last dose of study drug to assess the status of adverse events and to determine if any new events have occurred.

Subjects with HCC will be followed every 2 months (± 1 week) for survival status until death, or until lost to follow-up/withdrawal of consent. Follow-up may be conducted by record review (including public records) and/or telephone contact with the subject, family or the subject's treating physician, as appropriate.

EDMS Doc. Number: 20012337 - 16445273

6. STUDY POPULATION

Men and women, over 18 years old, with advanced NHL, MM, or advanced unresectable solid tumors, including subjects who have progressed on (or not been able to tolerate) standard therapy or for whom no standard anticancer therapy exists.

6.1. Number of Subjects and Sites

In Part A, approximately 20 to 40 subjects will be enrolled at two US sites and possibly some sites in Europe.

In Part B, approximately 230 subjects will be enrolled at approximately 15 sites in the US and Europe.

6.2. Inclusion Criteria

For both the dose escalation and dose expansion parts of this protocol:

1. Understand and voluntarily sign an informed consent document prior to any study related assessments/procedures are conducted
2. Men and women, 18 years or older, with histologically or cytologically-confirmed, advanced NHL, MM, or advanced unresectable solid tumors including subjects who have progressed on (or not been able to tolerate) standard anticancer therapy or for whom no standard anticancer therapy exists
3. ECOG PS of 0 or 1 for subjects with solid tumors, and 0 – 2 for hematologic malignancies
4. Subjects must have the following laboratory values:
    - Absolute Neutrophil Count (ANC) ≥ 1.5 x 10^9/L
    - Hemoglobin (Hgb) ≥ 9 g/dl
    - Platelets (plt) ≥ 100 x 10^9/L
    - Potassium within normal limits or correctable with supplements
    - AST/SGOT and ALT/SGPT ≤ 2.5 x Upper Limit of Normal (ULN) or ≤ 5.0 x ULN if liver tumor is present
    - Serum bilirubin ≤ 1.5 x ULN or ≤ 2 x ULN if liver tumor is present
    - Serum creatinine ≤ 1.5 x ULN or 24-hour clearance ≥ 50 mL/min
    - Negative serum or urine pregnancy test within 48 hours before starting study treatment in females of childbearing potential
5. Able to adhere to the study visit schedule and other protocol requirements

For the dose expansion part (Part B) of this protocol:

1. Retrieval of FFPE archival tumor tissue, either in tumor blocks or sectioned/mounted specimens for gene mutation and/or IHC biomarker assay for all tumors except MM.

EDMS Doc. Number: 20012337 - 16445273

Only in exceptional circumstances may an exemption waiver be granted by the Sponsor for other tumor types.

2. Satisfactory Screening biopsy for gene mutation and/or IHC biomarker assay for accessible tumors for all tumors except NSCLC and NET (optional), and GBM.
3. Histologically-confirmed tumors of the following types, all with measurable disease. Type-specific criteria are in addition to, or supersede, above criteria where applicable:
   a. Non-small cell lung cancer (NSCLC)
   b. Glioblastoma multiforme (GBM) or gliosarcoma (Louis, 2007), excluding WHO Grade IV oligoastrocytoma:
      - Has received prior treatment including radiation and/or chemotherapy, with radiation completed > 12 weeks prior to Day 1
      - Planned salvage surgical tumor resection on Day 15 ± 7 days, anticipated to yield ≥ 200 mg tumor tissue
      - No prior or scheduled Gliadel® wafer implant unless area of assessment and planned resection is outside the region previously implanted
      - No prior interstitial brachytherapy or stereotactic radiosurgery unless area of assessment and planned resection is outside the region previously treated
      - No enzyme-inducing anti-epileptic drugs (EIAED) such as carbamazepine, phenytoin, phenobarbital, or primidone within 14 days before Day 1
      - Able to undergo repeated magnetic resonance imaging (MRI) scans
      - Availability of adequate FFPE archival tumor material (for PD biomarkers)
   c. Hepatocellular carcinoma (HCC):
      - Plt count ≥ 60 x 10^9/L if portal hypertension is present
      - Child-Pugh score of less than 7 (ie, class A liver function or better) (Appendix D; Section 18.4; Pugh, 1973)
      - At least 4 weeks from last dose of α-interferon and/or ribivirin
      - At least 4 weeks from prior percutaneous ethanol injection, radiofrequency ablation, transarterial embolization, or cryotherapy with documentation of progressive or recurrent disease.
   d. Neuroendocrine tumor (NET) of non-pancreatic origin:
      - Locally unresectable or metastatic well differentiated, low (grade 1) or intermediate (grade 2), non-pancreatic NET or NET of unknown primary origin
      - Pancreatic NET, pheochromocytomas, paragangliomas, adenocarcinoid and goblet carcinoid tumors, and poorly differentiated, high grade (eg, small or large cell) tumors are excluded (Rindi, 2007)

EDMS Doc. Number: 20012337 - 16445273

- Symptomatic endocrine-producing tumors and nonfunctional tumors are both allowed
- Agreement to concurrent therapy with somatostatin analogs (SSA)
- Evidence of radiologic disease progression ≤ 12 months prior to Cycle 1, Day 1
- No receptor-targeted radiolabeled therapy ≤ 3 months prior to Cycle 1, Day 1
- No liver-directed therapy ≤ 4 weeks prior to Cycle 1, Day 1 unless a site of measurable disease other than the treated lesion is present
- Screening and on-study tumor biopsies are optional in this cohort. Archival tumor collection should be requested, but is not mandatory

e. Hormone receptor-positive breast cancer (HRPBC):

- Unresectable locally advanced or metastatic carcinoma of the breast
- ER positive, and HER2/neu negative (0 or 1+), tumor
- Measurable disease according to RECIST v1.1
- At least one year of aromatase inhibitor therapy in the adjuvant setting, or 6 months of aromatase inhibitor therapy for metastatic disease
- Bisphosphonates or denusomab are allowed in stable doses
- Cohort may be expanded to enroll a minimum of 5 subjects each with tumors containing PIK3CA mutations.

f. Multiple Myeloma (MM):

- Measurable levels of myeloma paraprotein in serum (> 0.5 g/dL) or urine (> 0.2 g excreted in a 24-hour collection sample)
- Absolute Neutrophil Count (ANC) ≥ 1.0 x 10^9/L
- Platelets (plt) ≥ 60 x 10^9/L in subjects in whom < 50% of bone marrow mononuclear cells are plasma cells or ≥ 30 x 10^9/L in subjects in whom ≥ 50% of bone marrow mononuclear cells are plasma cells

g. Diffuse large B-cell lymphoma (DLBCL):

- Histologically proven diffuse large B-cell non-Hodgkin's lymphoma
- Platelets (plt) ≥ 60 x 10^9/L for subjects in whom < 50% of bone marrow mononuclear cells are lymphoma cells, or ≥ 30 x 10^9/L for subjects in whom ≥ 50% of bone marrow mononuclear cells are lymphoma cells
- At least 4 weeks from last dose of therapeutic glucocorticosteroids. Adrenal replacement doses of glucocorticosteroids (up to the equivalent of 10 mg daily prednisone) are allowed.

EDMS Doc. Number: 20012337 - 16445273

6.3. Exclusion Criteria

For both the dose escalation and dose expansion parts of this protocol:

1. Symptomatic central nervous system metastases (excluding GBM, per Inclusion Criterion 6c). Subjects with brain metastases that have been previously treated and are stable for 6 weeks are allowed.
2. Known acute or chronic pancreatitis
3. Subjects with any peripheral neuropathy ≥ NCI CTCAE grade 2
4. Subjects with persistent diarrhea or malabsorption ≥ NCI CTCAE grade 2, despite medical management
5. Impaired cardiac function or clinically significant cardiac diseases, including any of the following
   - LVEF < 45% as determined by MUGA scan or ECHO
   - Complete left bundle branch, or bifascicular, block
   - Congenital long QT syndrome
   - Persistent or clinically meaningful ventricular arrhythmias or atrial fibrillation
   - QTcF > 460 msec on screening ECG (mean of triplicate recordings)
   - Unstable angina pectoris or myocardial infarction ≤ 3 months prior to starting CC-223
   - Other clinically significant heart disease such as congestive heart failure requiring treatment or uncontrolled hypertension (blood pressure ≥ 160/95 mmHg)
6. Subjects with diabetes on active treatment or subjects with either of the following:
   a. Fasting blood glucose ≥ 126 mg/dL (7.0 mmol/L), or
   b. HbA1c ≥ 6.5%
7. Other concurrent severe and/or uncontrolled concomitant medical conditions (eg, active or uncontrolled infection) that could cause unacceptable safety risks or compromise compliance with the protocol
8. Prior systemic cancer-directed treatments or investigational modalities ≤ 5 half lives or 4 weeks, whichever is shorter, prior to starting study drug or who have not recovered from side effects of such therapy. Subjects must have recovered from any effects of recent radiotherapy that might confound the safety evaluation of study drug.
9. Subjects who have undergone major surgery ≤ 2 weeks prior to starting study drug or who have not recovered from side effects of such therapy
10. Women who are pregnant or breast feeding. Adults of reproductive potential not employing two forms of birth control
    a. Females of childbearing potential must agree to use two adequate forms of contraception methods simultaneously (one must be non-hormonal) from the time of giving informed consent until 28 days after the last dose of CC-223. Females of

EDMS Doc. Number: 20012337 - 16445273

child-bearing potential, defined as sexually mature women who have not undergone a hysterectomy or bilateral oophorectomy, or who have not been naturally postmenopausal (ie, who have not menstruated at all) for at least 24 consecutive months

b. Males with partners who are female with child-bearing potential must agree that they or their partners will use at least two effective contraceptive methods (including one barrier method) when engaging in reproductive sexual activity throughout the study, and will avoid conceiving for 28 days after the last dose of CC-223.

11. Subjects with known HIV infection
12. Known chronic hepatitis B or C virus (HBV/HCV) infection, unless comorbidity in subjects with HCC
13. Any significant medical condition, laboratory abnormality, or psychiatric illness that would prevent the subject from participating in the study
14. Any condition including the presence of laboratory abnormalities, which places the subject at unacceptable risk if he/she were to participate in the study
15. Any condition that confounds the ability to interpret data from the study

For the dose expansion part (Part B) of this protocol:

1. Concurrent active second malignancy for which the patient is receiving therapy, excluding non-melanomatous skin cancer or carcinoma in situ of the cervix.

EDMS Doc. Number: 20012337 - 16445273

7. DESCRIPTION OF STUDY TREATMENTS

7.1. Description of Investigational Product(s)

7.1.1. Physical properties

CC-223 is a fine white to off-white powder with a melting point of 200.7°C.

7.1.2. Formulation

Celgene Corporation will supply CC-223 in appropriate strengths containing only the active pharmaceutical ingredient in reddish-brown gelatin capsules for oral administration. No other excipients are used in the product capsules.

7.1.3. Storage Conditions

Store as directed on package label.

7.2. Treatment Administration and Schedule

CC-223 is administered orally, in an uninterrupted once-daily schedule with no rest period between cycles. Each dose will be taken in the morning. On clinic visit days, CC-223 will be administered in the clinic after any predose tests have been completed. Food will be taken after all fasting tests have been completed (3 hours after dosing on Day 8). In cases where troublesome GI symptoms, fatigue or other symptoms persist beyond the end of Cycle 1, dosing may be moved to the end of day. CC-223 may be taken up to 12 hours late if dosing has been delayed on a single day; otherwise that days' dose should be omitted

In Part A, CC-223 will be administered once daily.

Doses will be administered in an escalating manner following satisfactory review of safety data from the lower doses. There will be a minimum of 30 days between dose escalations. Within each cohort, enrollment will be staggered so that there is a minimum of 24 hours between Cycle 1 Day 1 for each subject.

A Safety Review Committee (SRC) will review the safety and PK-PD data at the end of Cycle 1 dosing for each cohort and determine whether dose escalation can occur, to confirm the dose level to be used in a subsequent cohort, and to determine the MTD. The SRC will also determine the dose level for Part B.

The SRC will comprise the Principal Investigators, the sponsor's study physician and safety physician, and the study manager. In addition, the study pharmacokineticist and the statistician may attend, if needed, and both internal and external experts may be consulted by the SRC, as necessary. The SRC will continue to review data periodically during Part B. The outcomes of SRC meetings will be minuted, and distributed by the study manager for approval by the SRC.

7.2.1. Definition of a Treatment Cycle

During the first cycle in Part A, each subject will be administered a single dose of CC-223 (Day -1), followed by a 48-hour observation and PK sample collection period, which is then

EDMS Doc. Number: 20012337 - 16445273

followed on Day 1 by daily dosing for 28 days (Cycle 1 = 30 days). If necessary for logistical reasons, Day 1 may be delayed by up to 4 days. Subjects may continue to receive CC-223 for as long as they derive benefit from treatment as judged by the Investigator. In subsequent cycles, subjects are treated in 28-day cycles with continuous dosing from Day 1 to 28 without rest between cycles.

In Part B, each cycle is 28 days with continuous daily dosing. Therapy may be discontinued when there is evidence of disease progression, but subjects can continue to receive CC-223 as long as the Investigator considers they are deriving benefit from treatment. Therapy will be discontinued if there is unacceptable toxicity or if the subject decides to withdraw from the study.

7.2.2. Starting Dose Level for Cycle 1

In Part A, a dose of 7.5 mg/day is selected as the starting dose (see Section 1.2 for details on the starting dose rationale).

In Part B, subjects may start CC-223 at the MTD and/or a lower dose level, based on safety, PK and PD evaluations from Part A; the starting dose was determined to be 45 mg QD. Enrollment of subjects will be stratified by tumor cohort.

Based on review of available study data as of November 30, 2012, which showed a high frequency of dose reductions to 30 mg QD in Part B, the starting dose for newly enrolled subjects in expanded dose cohorts in Part B is reduced to 30 mg QD.

7.2.3. Dose Escalation Levels

In Part A, a modified accelerated titration design (Simon, 1997) will be used to identify initial toxicity. Each subject will be administered a single dose, followed by a 48-hour observation and PK sample collection period, followed by daily dosing for an additional 28 days (30 days total in this cycle). Subsequently, subjects will be treated in 28-day cycles with continuous dosing without treatment rests. All subjects will be treated and observed for at least 30 days after the first dose of CC-223 before the dose level is escalated in another cohort. Intra-subject dose escalation is not permitted unless approved by the SRC.

During the accelerated course, initial cohorts of one subject will be given CC-223 in dose increments of 100% (ie, doubling the dose level each time) until the first instance of first-course grade 2 or higher toxicity suspected to be CC-223-related, at which point the accelerated part will be terminated and that particular cohort will be expanded to 6 subjects. Even in the absence of such toxicity, additional subjects may be evaluated within a dose cohort if recommended by the SRC to adequately evaluate the safety or treatment effects of CC-223. Subsequent to grade 2 or higher CC-223-related toxicity occurring, a standard escalation dosing schedule using approximately 50% dose increments and 6 subjects per cohort will be used, unless the SRC considers that based on safety, tolerability and/or PK data, an intermediate dose escalation (eg, 25%) should be evaluated.

7.2.4. Definition of an Evaluable Subject

All subjects who receive at least one dose of CC-223 will be evaluable for safety.

EDMS Doc. Number: 20012337 - 16445273

In Part A, a subject evaluable for DLT is defined as one who, in the first 30 days after Cycle 1 dosing began, either (a) received at least 24 of the planned 29 doses of CC-223 at the cohort-specified dose and has sufficient data for safety evaluation by the SRC, or (b) experienced study drug-related DLT. Non-evaluable subjects will be replaced in the dosing cohort.

In Part B, an efficacy evaluable subject is defined as one who received at least one cycle of CC-223 and had a baseline and at least one post-baseline efficacy assessment.

7.2.5. Definition of Non-tolerated dose (NTD)

The non-tolerated dose is defined as the dose level at which 2 evaluable subjects in any dose cohort experience DLT.

7.2.6. Definition of Maximum Tolerated Dose (MTD)

Once the NTD is identified, dose escalation will be stopped. The MTD is defined as the last dose level below the NTD with 0 or 1 (out of 6) evaluable subjects experiencing DLT during cycle 1. An intermediate dose (ie, one between the NTD and the last dose level before the NTD) may be evaluated in order to determine the MTD more precisely.

7.2.6.1. Definition of Dose-Limiting Toxicity (DLT)

National Cancer Institute Common Terminology Criteria for Adverse Events (NCI CTCAE) Version 4, 2009 are used to grade adverse events. Dose-limiting toxicities are described below:

- A clinically relevant AE that is suspected to be related to CC-223 and that commences within 30 days of first dose (Cycle 1) and is ≥ grade 3 EXCEPT for:
  - Alopecia
  - Grade 3 rash of the acneiform or maculopapular type of not more than 4 days duration (with optimal medical management)
  - Grade 3 diarrhea or vomiting lasting less than 72 hours (with optimal medical management)
  - Repeated occurrence of CTCAE grade 3 hyperuricaemia (ULN to 10 mg/dL with physiologic consequences) in a subject with grade 3 hyperuricemia at baseline.
  - Hyperglycemia, hematologic and LFT abnormalities as defined below
- Clinically relevant laboratory abnormality that is suspected to be related to CC-223 and that commences within 30 days of first dose (Cycle 1) and is ≥ grade 3.
- Grade 2 (>160 mg/dL) fasting hyperglycemia lasting > 14 days despite optimal medical treatment, or fasting grade 3 or greater (glucose > 250 mg/dL) lasting > 4 days despite optimal medical treatment. Further information regarding the monitoring and management of hyperglycemia is given in Appendix C, Section 18.3.1 Guidance for Managing Hyperglycemia.
- Hematological toxicities as follows:
  - Any febrile neutropenia

EDMS Doc. Number: 20012337 - 16445273

  - Grade 4 neutropenia lasting > 7 days
  - Grade 4 thrombocytopenia lasting > 7 days
  - Any grade 3/4 thrombocytopenia with clinically significant bleeding
- Grade 4 liver function tests (LFTs) will be considered DLT irrespective of underlying attribution. However, grade 3 LFTs due to disease progression in the liver will not be considered DLT.
- Any adverse event suspected to be CC-223 related and necessitating dose reduction during Cycle 1 (Part A only).

Isolated laboratory changes without associated clinical signs or symptoms may not be included in this definition. These findings will be discussed and reviewed by the SRC.

7.2.6.2. Criteria for Dose Escalation in the Next Cohort of Subjects

After the accelerated phase of Part A (beyond the single subject cohorts), cohorts will consist of 6 subjects. Enrollment of the 6 subjects must be staggered by at least 24 hours for each subject. The decision criteria for dose escalation in any cohort are:

- If 0 or 1 of at least 6 evaluable subjects experience DLT within 30 days during Cycle 1, dose escalation to the next higher dose level may occur.
- If $\geq$ 2 of 6 evaluable subjects experience a DLT within 30 days during Cycle 1, any further recruitment will cease and the dose will be defined as the NTD.
- SRC will determine if additional subjects need to be enrolled at the previous dose to have 6 evaluable subjects in order to define MTD, or whether an intermediate dose level will be explored in up to 6 newly enrolled subjects.

The number of cohorts depends on incidence of DLT. A subject may experience more than 1 DLT. Dose escalation decisions are based on the number of subjects experiencing DLT events.

7.2.6.3. Definition of Stopping Criteria for Dose Escalation

Dose escalation stops when $\geq$2 subjects at a dose level experience DLT.

7.2.7. Permitted Study Drug Adjustments

In Parts A and B, dose reductions are permitted in any cycle, including Cycle 1. Dose reductions that occur in Cycle 1 during Part A will constitute DLT, but subjects will be allowed to continue on study drug at the reduced dose. National Cancer Institute Common Terminology Criteria for Adverse Events (NCI CTCAE) Version 4, 2009 will be used to grade adverse events.

When a dose reduction is indicated, the next lower dose level will be selected. Two dose reductions are allowed. For the starting dose level (7.5 mg) in Part A, reductions will be in 2.5 mg decrements. In Part B for subjects starting at 45 mg QD, dose reductions to 30 mg and 15 mg QD are permitted; for those starting at 30 mg QD, the dose reductions are 20 mg QD and 15 mg QD. If any subject continues to experience unacceptable toxicity after 2 dose reductions in Part A, CC-223 will be discontinued permanently. In Part B subjects may dose reduce up to 2 levels

EDMS Doc. Number: 20012337 - 16445273

and increase again if clinically appropriate; subsequent dose reductions are permitted in the event of recurrent toxicity but, in such circumstances, it is not permitted to reescalate the dose again.

With (optional) subject consent, blood should be drawn for PK assessments predose (< 15 mins prior to dosing) and 1 hour ±10 mins, 1.5 hours ± 10 mins, 3 hours ± 10 mins and 5 hours ± 15 mins post dose 7 – 14 days following dose reduction in order to evaluate intrasubject study drug PK.

7.2.7.1. Criteria for Dose Reduction:

Any of the following will require dose reduction:

- A clinically relevant AE or laboratory abnormality that is suspected to be related to CC-223 and that is ≥ grade 3 EXCEPT for:
  - Alopecia
  - Grade 3 rash of the acneiform or maculopapular type of not more than 4 days' duration (with optimal medical management)
  - Grade 3 diarrhea or vomiting lasting less than 72 hours (with optimal medical management)
  - Repeated occurrence of CTCAE grade 3 hyperuricaemia (ULN to 10 mg/dL with physiologic consequences) in a subject with a history of grade 3 hyperuricemia.
  - Grade ≥ 3 hyperglycemia of not more than 4 days' duration with optimal medical management
- Any of the following hematological events:
  - Any febrile neutropenia
  - Grade 4 neutropenia lasting > 7 days
  - Grade 4 thrombocytopenia lasting > 7 days
  - Any grade 3/4 thrombocytopenia with clinically significant bleeding

Once dosage has reduced, it can be escalated when toxicity reaches ≤ grade 1. If toxicity recurs at the higher dose, the dose will be reduced a second time but no re-escalation is then permitted.

7.2.7.2. Criteria for Dose Increase

Intrasubject dose escalation beyond the dose initially assigned to a subject is not permitted in Cycle 1. Those continuing to take CC-223 longer term may, following approval by the SRC, have the dose level increased providing the alternative dose level has been shown to be well tolerated by at least one cohort of other subjects in this study.

For subjects in Part B starting at 30 mg QD, dose escalation to 45 mg QD is not allowed.

EDMS Doc. Number: 20012337 - 16445273

7.2.8. Treatment Delay

7.2.8.1. Treatment Delay for Adverse Events

Treatment may be delayed up to 4 weeks until treatment-related toxicity (excluding alopecia) reaches either ≤ grade 1 or baseline levels. Treatment may restart either at the same, or a reduced, dose level, at the Investigator's discretion or as described in Section 7.2.7. In Part A during Cycle 1 a treatment interruption of > 5 days for reasons other than DLT will make a subject non-evaluable for DLT and necessitate replacement of that subject in the dosing cohort.

7.2.8.2. Treatment Delay for Postoperative GBM subjects

Treatment may be delayed for up to 4 weeks, until a subject has recovered from salvage tumor resection and is on a stable or decreasing dose of glucocorticoids for at least 5 days. Longer delays require approval by the Sponsor. During the postoperative period, but with the exception of AE reporting, C1D22 procedures may be omitted if subjects are not taking CC-223. Postoperative concomitant medications/procedures exclusively for surgical support and unrelated to study medication need not be documented on the eCRF.

7.3. Method of Treatment Assignment

All subjects enrolled on this study will receive treatment with CC-223. Dose level assignment in Part A will be as described in Section 7.2.3. In Part B, subjects may start CC-223 at the MTD and/or a lower dose level, based on safety, PK and PD evaluations from Part A. Treatment allocation in Part B will be prospectively stratified by tumor cohort.

7.4. Packaging and Labeling

CC-223 capsules are packaged in high density polyethylene (HDPE) bottles fitted with an induction seal and child-resistant plastic closure.

The IP will be labeled per local regulations.

7.5. Investigational Product Accountability and Disposal

Celgene will instruct the Investigator on the return, disposal and/or destruction of IP and/or medical device materials if applicable.

7.6. Investigational Product Compliance

Only the pharmacist or Investigator's designee will dispense the study drug. A record of the number of capsules dispensed to and taken by each subject must be maintained. The pharmacist or Investigator's designee will document the dose dispensed in the appropriate study records.

Subjects will use weekly diary cards to record their daily self-administration of CC-223 at home. These will be reviewed by study staff each time the subject visits the clinic. Entries will be clarified, as necessary, so that appropriate information can be captured on the eCRFs.

EDMS Doc. Number: 20012337 - 16445273

9. STATISTICAL ANALYSES

9.1. Overview

The primary objectives of this Phase 1/2 study are to determine the safety, tolerability, NTD and MTD of CC-223 when administered orally to adult subjects with advanced solid tumors, NHL and MM and to determine the PK characteristics of oral CC-223. The secondary objectives are to evaluate the degree of inhibition achievable with CC-223 for key markers associated with mTOR pathways and, in Part B, to explore the antitumor activity of CC-223 at selected dose levels/regimens by tumor type.

In the following, statistical analyses will be performed by study phase, dose level, dosing regimen and tumor type as needed or applicable.

9.2. Study Population Definitions

The study population definitions are as follows:

- Intent-to-Treat (ITT) Population – all subjects who take at least one dose of CC-223.
- Safety Population – all subjects who take at least one dose of CC-223, which is the same as ITT population for this study.
- Efficacy Evaluable (EE) Population – all ITT subjects who meet eligibility criteria, complete at least one cycle of CC-223, and have baseline and at least one post-baseline efficacy assessment.

9.3. Sample Size and Power Considerations

During Part A of the study, a modified accelerated titration design (Simon, 1997) will be used for dose escalation as described in Section 3.1, and approximately 20 to 40 subjects will be enrolled.

After the MTD is determined from the Part A, Part B will be opened to enroll approximately 230 additional subjects. Tumor types selected for Part B include NSCLC, GBM, HCC, NET, HRPBC, DLBCL, and MM. Up to 40 subjects will be enrolled in each tumor type.

In Part B within each tumor type, enrollment and tumor response rate monitoring will be based on Wald's sequential analysis (Wald, 1945). The Wald's sequential design gives the futility bound and efficacy bound. Since the objective of Part B is to identify potentially promising tumor type(s), we will stop the enrollment because of futility, but may not stop the enrollment early because of efficacy in order to estimate the response rate more precisely. Assuming the target response rate being 20% for the tumor type of interest, based on the sequential design, with 80% power to identify the group with 20% or more response rate at 5% significance level when the response rate is at 10%, the enrollment will be stopped for futility when there is no responder out of up to 14 evaluable subjects.

Subject enrollment will be curtailed when a total of approximately 230 evaluable subjects have been enrolled or when up to 40 evaluable subjects are evaluable in each tumor type. For Part B as a whole, sample sizes are not based on statistical calculation but rather on clinical empirical and practical considerations traditionally used for Phase 1/2 studies of this kind.

EDMS Doc. Number: 20012337 - 16445273

9.4. Background and Demographic Characteristics

In Part A, the baseline characteristics of subjects enrolled in each cohort by CC-223 dose level will be summarized. In Part B, the baseline characteristics of subjects will be summarized by tumor type. The age, weight, height and other continuous demographic and baseline variables will be summarized using descriptive statistics. Performance status, gender, race and other categorical variables will be summarized with frequency tabulations. Medical history data will be summarized using frequency tabulations by system organ class and preferred term.

9.5. Subject Disposition

Subject disposition (analysis population allocation, entered, discontinued, along with primary reason for discontinuation) will be summarized using frequency and percent for both treatment and follow-up phases. A summary of subjects enrolled by site will be provided. Protocol deviations will be summarized using frequency tabulations.

9.6. Efficacy Analysis

All treated subjects will be included for efficacy analysis. The efficacy variables of primary interest are tumor response at the end of treatment. Tumor response will be based on RECIST 1.1 for solid tumors, IWG for NHL/DLBCL, or IURC for MM (Appendix A, Section 18.1), and assessed by investigator. For GBM, RANO criteria (Appendix A, Section 18.1) will be used for tumor response, using the post resection MRI scan as the baseline. Given the difficulty in assessing tumor response following salvage surgery, the primary efficacy endpoint for GBM will be the proportion of subjects progression-free at 6 months from Day 1 relative to efficacy evaluable subjects in the GBM type. Subjects will be evaluated for tumor response on completion of Cycle 2, 4, 6, and so on. A descriptive analysis of evidence of anti-tumor activity will be provided based on clinical and radiographic assessments by the investigator, which includes assessment of target lesion, non-target lesion, new lesion and overall response.

The efficacy variable of focus for Part A will be best overall response. Other preliminary efficacy variables will be summarized using frequency tabulations for categorical variables or descriptive statistics for continuous variables.

For Part B, efficacy variables to be analyzed include tumor response at the end of treatment, the proportion of subject alive and progression-free, and duration of response. Efficacy variables will mature when last subject of a treatment arm or cohort have withdrawn from the study or completed 6 cycles.

Progression Free Survival rates will be computed using the Kaplan-Meier estimates. Duration of response will also be reported in subjects who respond, using tumor specific evaluation criteria. Two-sided 90% CIs of the Response Rate (RR), Disease Control Rate (DCR) and of the Progression Free Survival (PFS) rate at time of each scheduled response assessment (ie, Cycles 2, 4, 6, etc.) will be provided by tumor type.

Other preliminary efficacy variables, including ECOG PS, PET, carcinoid/NET-specific symptom outcomes, etc., will be summarized using frequency tabulations for categorical variables or descriptive statistics for continuous variables. The Kaplan-Meier estimate for overall survival (HCC cohort) will be provided.

EDMS Doc. Number: 20012337 - 16445273

Full details on the efficacy analysis will be given in the SAP.

Once all NET subjects complete long-term follow-up in the study, the PFS for NET subjects will be updated using the Kaplan-Meier estimates based on all data before and after the data cut-off date. The assessments of serum hormones, carcinoid/NET-specific symptom outcomes, carcinoid symptom relief, etc. in these subjects will be summarized using the residual data after the data cut-off date.

9.7. Safety Analysis

All subjects who receive at least one dose of study medication will be included in the safety analyses. Adverse events, vital sign measurements, physical exam findings, clinical laboratory information, ECG interpretations, LVEF assessments, concomitant medications and procedures will be tabulated and summarized by study phase, dose level, dosing regimen and tumor type, as appropriate. ECGs will be reviewed centrally.

During the Part A Cycle 1, DLTs and all available safety information will be reviewed on an ongoing basis by the Investigators and sponsor and summarized at the conclusion of each dose level. After completion of each dose cohort, the SRC will review the summarized data to determine the next step. Complete safety data for the Part A Cycle 1 will be summarized when all subjects have completed the first 30-day cycle.

Adverse events observed will be classified using the MedDRA classification system. The severity of the toxicities will be graded according the NCI CTCAE v 4.0 whenever possible.

The frequency of adverse events will be tabulated by MedDRA System Organ Class and Preferred Term. In the by-subject analysis, a subject having the same event more than once will be counted only once. Adverse events will be summarized by NCI CTCAE grade. Adverse events leading to discontinuation from treatment, events classified as NCI CTCAE grade 3 or higher, study-drug-related events, and serious adverse events will be tabulated and listed separately. By-subject listings of all adverse events, serious adverse events, and their attributes will be provided. Frequency of these events will be summarized with descriptive statistics.

Clinical laboratory data will be summarized. Lab data will be graded according to NCI CTCAE version 4.0 criteria wherever possible. The frequencies of the worst severity grade observed during treatment will be displayed in cross-tabulations by screening status.

Vital signs, ECG data and LVEF data will be summarized by cross-tabulations presenting normal and abnormal values by number of subjects at pre- and post-study drug initiation.

Graphical displays will be provided where useful in the interpretation of results.

9.8. Interim Analysis

No interim analysis is planned.

9.9. Assessment of Pharmacokinetics

Pharmacokinetic measures are incorporated in the study to assess the extent of exposure to CC-223 and M1, and to explore the relationship between CC-223 and PD effects. Blood and urine samples for PK will be collected at selected visits from all subjects. The following PK

EDMS Doc. Number: 20012337 - 16445273

measurements for CC-223 and/or M1 will be determined from plasma concentration vs. time profile data:

- Area under the plasma concentration time-curve after a dose of CC-223 (AUC)
- Peak (maximum) plasma concentration (C_{max})
- Terminal half-life ($T_{1/2}$)
- Time to maximum plasma concentration (T_{max})
- Total body Clearance (CL/F)
- Apparent volume of distribution (Vz/F)
- Amount of urinary excretion (Ae) and Renal clearance (CL_r)

Drug concentration in tumor tissue will be determined when sufficient amounts of biopsy tissue are available. Descriptive statistics (N, mean, SD, coefficient of variation [CV%], standard error [SE], geometric mean, geometric CV%, median, min, and max) will be provided for all data. Results will be presented in tabular and graphic forms as appropriate.

9.10. Assessment of Pharmacodynamics

The PD measurements are incorporated in this study to evaluate target inhibition of mTORC1 and mTORC2 pathways, the consequences of such inhibition, and PK/PD relationships. In Parts A and B, biomarker analysis will involve measuring pAKT (mTORC2) in protein lysates derived from isolated platelets. Levels of p4EB-P1 and pS6RP (mTORC1), and pAKT (mTORC2), will be measured by flow cytometry using whole blood samples. Likewise, in Parts A and B, pAKT, p4EB-P1, pS6, Ki67 and/or other relevant markers to assess CC-223 activity will be measured in serial tumor biopsies from subjects with accessible disease when possible. The changes of each biomarker will be determined by comparing the levels of biomarkers in pre- and post-treatment samples and, where possible, correlate these with drug exposure in blood, and tissue if available, and tumor response over time. Full details of all statistical analyses and modeling for these outcomes will be described in the statistical analysis plan and final study report.

9.11. Evaluation of Potential Predictive Biomarkers

Analysis of blood and/or tumor derived archival tissue, protein lysates, RNA or DNA for components in the PI3K/AKT/mTOR pathway, the p53 family members, and other relevant proteins or genetic variants, including but not limited to LKB1 mutation status, will be incorporated in this study to identify potential predictive biomarkers that correlate with efficacy.

9.12. Methods and Timing of PK/PD Sampling

Sample collection kits and detailed instructions for PK and PD biomarker sample collection, processing, storage, shipping and handling will be provided to the sites upon study initiation.

Plasma and urine CC-223 and M1 will be measured using validated liquid chromatography-mass spectrometry methods (LC-MS/MS).

EDMS Doc. Number: 20012337 - 16445273

10. ADVERSE EVENTS

10.1. Monitoring, Recording and Reporting of Adverse Events

An adverse event (AE) is any noxious, unintended, or untoward medical occurrence that may appear or worsen in a subject during the course of a study. It may be a new intercurrent illness, a worsening concomitant illness, an injury, or any concomitant impairment of the subject's health, including laboratory test values (as specified by the criteria below), regardless of etiology. Any worsening (ie, any clinically significant adverse change in the frequency or intensity of a pre-existing condition) should be considered an AE. A diagnosis or syndrome should be recorded on the AE page of the eCRF rather than the individual signs or symptoms of the diagnosis or syndrome.

An overdose, accidental or intentional, whether or not it is associated with an AE, or abuse, withdrawal, sensitivity or toxicity to an investigational product should be reported as an AE. If an overdose is associated with an AE, the overdose and AE should be reported as separate terms.

All subjects will be monitored for AEs during the study. Assessments may include monitoring of any or all of the following parameters: the subject's clinical symptoms, laboratory, pathological, radiological or surgical findings, physical examination findings, or other appropriate tests and procedures.

All AEs will be recorded by the Investigator from the time the subject signs informed consent to 28 days after the last dose of IP. AEs and serious adverse events (SAEs) will be recorded on the AE page of the eCRF and in the subject's source documents. All SAEs must be reported to Celgene Drug Safety immediately (ie, within 24 hours of the Investigator's knowledge of the event) by facsimile, or other appropriate method, using the SAE Report Form, or approved equivalent form.

10.2. Evaluation of Adverse Events

A qualified Investigator will evaluate all AEs as to:

10.2.1. Seriousness

A serious adverse event (SAE) is any AE occurring at any dose that:

- Results in death;
- Is life-threatening (ie, in the opinion of the Investigator, the subject is at immediate risk of death from the AE);
- Requires hospitalization or prolongation of existing hospitalization (hospitalization is defined as an insubject admission, regardless of length of stay);
- Results in persistent or significant disability/incapacity (a substantial disruption of the subject's ability to conduct normal life functions);
- Is a congenital anomaly/birth defect;
- Constitutes an important medical event.

EDMS Doc. Number: 20012337 - 16445273

Important medical events are defined as those occurrences that may not be immediately life threatening or result in death, hospitalization, or disability, but may jeopardize the subject or require medical or surgical intervention to prevent one of the other outcomes listed above. Medical and scientific judgment should be exercised in deciding whether such an AE should be considered serious.

Events **not considered** to be SAEs are hospitalizations for:

- A standard procedure for protocol therapy administration. However, hospitalization or prolonged hospitalization for a complication of therapy administration will be reported as an SAE.
- Routine treatment or monitoring of the studied indication not associated with any deterioration in condition.
- The administration of blood or platelet transfusion as routine treatment of studied indication. However, hospitalization or prolonged hospitalization for a complication of such transfusion remains a reportable SAE.
- A procedure for protocol/disease-related investigations (eg, surgery, scans, endoscopy, sampling for laboratory tests, bone marrow sampling). However, hospitalization or prolonged hospitalization for a complication of such procedures remains a reportable SAE.
- Hospitalization or prolongation of hospitalization for technical, practical, or social reasons, in absence of an AE.
- A procedure that is planned (ie, planned prior to starting of treatment on study); must be documented in the source document and the eCRF. Hospitalization or prolonged hospitalization for a complication remains a reportable SAE.
- An elective treatment of a pre-existing condition unrelated to the studied indication.
- Emergency outsubject treatment or observation that does not result in admission, unless fulfilling other seriousness criteria above.

If an AE is considered serious, both the AE page/screen of the eCRF and the SAE Report Form must be completed.

For each SAE, the Investigator will provide information on severity, start and stop dates, relationship to IP, action taken regarding IP, and outcome.

10.2.2. Severity

For both AEs and SAEs, the Investigator must assess the severity of the event as described by CTCAE v4.

Grade 1: Mild; asymptomatic or mild symptoms; clinical or diagnostic observations only; intervention not indicated.

Grade 2: Moderate; minimal, local or noninvasive intervention indicated; limiting age-appropriate instrumental self care activities of daily living (ADL).

EDMS Doc. Number: 20012337 - 16445273

Grade 3: Severe or medically significant but not immediately life-threatening; hospitalization or prolongation of hospitalization indicated; disabling; limiting self care ADL.

Grade 4: Life-threatening consequences; urgent intervention indicated.

Grade 5: Death related to AE.

The term “severe” is often used to describe the intensity of a specific event (as in mild, moderate or severe myocardial infarction); the event itself, however, may be of relatively minor medical significance (such as severe headache). This criterion is *not* the same as “serious” which is based on subject/event *outcome* or *action* criteria associated with events that pose a threat to a subject’s life or functioning.

Seriousness, not severity, serves as a guide for defining regulatory obligations.

10.2.3. Causality

The Investigator must determine the relationship between the administration of IP and the occurrence of an AE/SAE as Not Suspected or Suspected as defined below:

Not Suspected:	The temporal relationship of the adverse event to IP administration makes **a causal relationship unlikely or remote**, or other medications, therapeutic interventions, or underlying conditions provide a sufficient explanation for the observed event.
Suspected:	The temporal relationship of the adverse event to IP administration makes **a causal relationship possible**, and other medications, therapeutic interventions, or underlying conditions do not provide a sufficient explanation for the observed event.

If an event is assessed as suspected of being related to a comparator, ancillary or additional IP that has not been manufactured or provided by Celgene, please provide the name of the manufacturer when reporting the event.

10.2.4. Duration

The Investigator will provide a record of the start and stop dates of the event.

10.2.5. Action Taken

The investigator will report the action taken with IP as a result of an AE or SAE, (eg, discontinuation or reduction of IP) as appropriate, and report if concomitant and/or additional treatments were given for the event.

All SAEs that have not resolved upon discontinuation of the subject’s participation in the study must be followed until recovered, recovered with sequelae, not recovered (death due to another cause) or death (due to the SAE).

10.3. Abnormal Laboratory Values

An abnormal laboratory result is considered to be an AE if the abnormality:

- results in discontinuation from the study;

EDMS Doc. Number: 20012337 - 16445273

- requires treatment, modification/ interruption of IP dose, or any other therapeutic intervention; or
- is judged to be of significant clinical importance.

Regardless of severity grade, only laboratory abnormalities that fulfill a seriousness criterion need to be documented as a serious adverse event.

If a laboratory abnormality is one component of a diagnosis or syndrome, then only the diagnosis or syndrome should be recorded on the AE page/screen of the eCRF. If the abnormality was not a part of a diagnosis or syndrome, then the laboratory abnormality should be recorded as the AE.

10.4. Pregnancy

10.4.1. Females of Childbearing Potential:

Pregnancies and suspected pregnancies (including a positive pregnancy test regardless of age or disease state) of a female subject occurring while the subject is on IP, or within 28 days of the subject's last dose of IP, are considered immediately reportable events. IP is to be discontinued immediately and the subject instructed to return any unused CC-223 to the Investigator. The pregnancy, suspected pregnancy, or positive pregnancy test must be reported to Celgene Drug Safety immediately by facsimile, or other appropriate method, using the Initial Pregnancy Report Form.

The female may be referred to her obstetrician-gynecologist.

The Investigator will follow the female subject until completion of the pregnancy, and must notify Celgene Drug Safety immediately about the outcome of the pregnancy (either normal or abnormal outcome) using the Follow-up Pregnancy Report Form as a follow-up to the initial pregnancy report.

If the outcome of the pregnancy was abnormal (eg, spontaneous or therapeutic abortion [any congenital anomaly detected in an aborted fetus must be documented], stillbirth, neonatal death, or congenital anomaly [including that in an aborted fetus]), the Investigator should report the abnormal outcome as an AE. If the abnormal outcome meets any of the serious criteria, it must be reported as an SAE to Celgene Drug Safety immediately by facsimile, or other appropriate method, within 24 hours of the Investigator's knowledge of the event using the SAE Report Form or approved equivalent form.

All neonatal deaths that occur within 28 days of birth should be reported, without regard to causality, as SAEs. In addition, any infant death after 28 days that the Investigator suspects is related to the in utero exposure to the IP should also be reported to Celgene Drug Safety immediately by facsimile, or other appropriate method, within 24 hours of the Investigator's knowledge of the event using the SAE Report Form, or approved equivalent form.

10.4.2. Male Subjects

Female partners of male subjects taking investigational product should be advised to call their healthcare provider immediately if they become pregnant, and male subjects should notify the Investigator.

EDMS Doc. Number: 20012337 - 16445273

10.5. Immediate Reporting of Serious Adverse Events

Any AE that meets any criterion for an SAE requires the completion of an SAE Report Form in addition to being recorded on the AE page/screen of the eCRF. All SAEs must be reported to Celgene Drug Safety immediately (ie, within 24 hours of the Investigator's knowledge of the event) by facsimile, or other appropriate method, using the SAE Report Form, or approved equivalent form. This instruction pertains to initial SAE reports as well as any follow-up reports.

The Investigator is required to ensure that the data on these forms is accurate and consistent. This requirement applies to all SAEs (regardless of relationship to IP) that occur during the study (from the time the subject signs informed consent to 28 days after the last dose of IP), and those made known to the Investigator at anytime thereafter that are suspected of being related to IP. SAEs occurring prior to treatment will be captured.

The SAE report should provide a detailed description of the SAE and include a detailed summary of the event on the last page of the SAE Report Form. If a subject died and an autopsy has been performed, copies of the autopsy report and death certificate are to be sent to Celgene Drug Safety as soon as these become available. Any follow-up data will be detailed in a subsequent SAE Report Form, or approved equivalent form, and sent to Celgene Drug Safety.

Where required by local legislation, the Investigator is responsible for informing the IRB/EC of the SAE and providing them with all relevant initial and follow-up information about the event. The Investigator must keep copies of all SAE information on file including correspondence with Celgene and the IRB/EC.

10.5.1. Safety Queries

Queries pertaining to SAEs will be communicated from Celgene Drug Safety to the site via facsimile or electronic mail. The response time is expected to be no more than five (5) business days. Urgent queries (eg, missing causality assessment) may be handled by phone.

10.6. Expedited Reporting of Adverse Events

For the purpose of regulatory reporting, Celgene Drug Safety will determine the expectedness of events suspected of being related to CC-223 based on the Investigator's Brochure.

For countries within the European Economic Area (EEA), Celgene or its authorized representative will report in an expedited manner to Regulatory Authorities and Ethics Committees concerned, suspected unexpected serious adverse reactions (SUSARs) in accordance with Directive 2001/20/EC and the Detailed Guidance on collection, verification and presentation of adverse reaction reports arising from clinical trials on investigational products for human use (ENTR/CT3) and also in accordance with country-specific requirements.

Celgene or its authorized representative shall notify the Investigator of the following information

- Any AE associated with the use of IP in this study or in other studies that is both serious and unexpected (ie, SUSAR);
- Any finding from tests in laboratory animals that suggests a significant risk for human subjects including reports of mutagenicity, teratogenicity, or carcinogenicity.

EDMS Doc. Number: 20012337 - 16445273

Where required by local legislation, the Investigator shall notify his/her IRB/EC promptly of these new serious and unexpected AE(s) or significant risks to subjects.

The Investigator must keep copies of all pertinent safety information on file including correspondence with Celgene and the IRB/EC. (See Section 14.3 for record retention information).

10.7. Celgene Drug Safety Contact Information:

For Local Drug Safety Affiliate Office contact information, please refer to the Serious Adverse Event Report Form Completion Guidelines or to the SAE Report Form.

EDMS Doc. Number: 20012337 - 16445273

11. DISCONTINUATIONS

The following events are considered sufficient reasons for discontinuing a subject from the treatment phase and/or from the study:

- Adverse Event(s)
- Disease Progression
- Withdrawal of consent
- Death
- Lost to follow up
- Protocol violation

The reason for discontinuation should be recorded in the eCRF and in the source documents.

EDMS Doc. Number: 20012337 - 16445273

17. REFERENCES

Baselga, J. Targeting the phosphoinositide-3 (PI3) kinase pathway in breast cancer. *Oncologist* 2011; 16 Suppl 1: 12-19.

CC-223 Investigator's Brochure, version 2: 14 November, 2011.

Cheson BD, Pfistner B, Juweid, ME, et al. Revised Response Criteria for Malignant Lymphoma. J Clin Oncol: 2007: (25) 579-586.

Contreras CM, Akbay EA, Gallardo TD, Haynie JM, et al. Lkb1 inactivation is sufficient to drive endometrial cancers that are aggressive yet highly responsive to mTOR inhibitor monotherapy. Dis Model Mech. 2010 Mar-Apr;3(3-4):181-93.

Ding L, Getz G, Wheeler DA, Mardis ER, et al. Somatic mutations affect key pathways in lung adenocarcinoma. Nature. 2008 Oct 23;455(7216):1069-75.

Durie BGM, Harousseau J-L, Miguel JS, et al. International uniform response criteria for multiple myeloma. Leukemia, 2006; (10) 10: 1-7.

Eisenhauer EA, Therasse P, Bogaerts J, et al. New response evaluation criteria in solid tumours: Revised RECIST guideline (version 1.1). European J Cancer; 2009; (45) 228–247.

Engelman JA. Targeting PI3K signalling in cancer: opportunities, challenges and limitations. Nat Rev Cancer. 2009 Aug;9(8):550-62.

Fan D, Ma C, Zhang H. The molecular mechanisms that underlie the tumor suppressor function of LKB1. Acta Biochim Biophys Sin. 2009: 41(2):97-107.

Feldman ME, Apsel B, Uotila A, Loewith R, Knight ZA, Ruggero D, et al. Active-site inhibitors of mTOR target rapamycin-resistant outputs of mTORC1 and mTORC2. PLoS Biol. 2009 Feb 10;7(2):e38.

Flockhart DA. Drug Interactions: Cytochrome P450 Drug Interaction Table. Indiana University School of Medicine (2007). http://medicine.iupui.edu/clinpharm/ddis/ (Accessed 29 Mar 2010.)

Gibbons JJ, Abraham RT, Yu K. Mammalian target of rapamycin: discovery of rapamycin reveals a signaling pathway important for normal and cancer cell growth. Semin Oncol. 2009 Dec;36 Suppl 3:S3-S17.

Goldberg PA, Roussel MG, Inzucchi SE. Clinical results of an updated insulin infusion protocol in critically ill patients. Diabetes Spectrum 2005;18:188-91.

Goldberg PA, Siegel MD, Sherwin RS, et al. Implementation of a safe and effective insulin infusion protocol in a medical intensive care unit. Diabetes Care 2004;27:461-467.

Higgins, M. J. and Baselga, J. "Targeted therapies for breast cancer." *J Clin Invest* 2011; 121(10): 3797-3803.

Isakoff, S. J., Engelman, J. A., et al. Breast cancer–associated PIK3CA mutations are oncogenic in mammary epithelial cells. *Can Res* 2005: 65(23): 10992-11000.

Ji H, Ramsey MR, Hayes DN, Fan C, McNamara K, Kozlowski P, et al. LKB1 modulates lung cancer differentiation and metastasis. Nature. 2007 Aug 16;448(7155):807-10.

Jiao Y et al. DAXX/ATRX, MEN1, and mTOR pathway genes are frequently altered in pancreatic neuroendocrine tumors, Science 2011; 331:1199-1203.

Kim DH, Sarbassov DD, Ali SM, King JE, Latek RR, Erdjument-Bromage H, Tempst P, Sabatini DM. mTOR interacts with raptor to form a nutrient-sensitive complex that signals to the cell growth machinery. Cell. 2002 Jul 26;110(2):163-75.

Louis DN, Ohgaki H, Wiestler OD, Cavenee WK, Burger PC, Jouvet A, Scheithauer BW, Kleihues P. The 2007 WHO classification of tumors of the central nervous system. Acta Neuropathol. 2007; 114: 97-109.

Pugh RN, Murray-Lyon IM, Dawson JL, Pietroni MC, Williams R (1973). Transection of the oesophagus for bleeding oesophageal varices. British Journal of Surgery. 1973; 60(8): 646–9.

Rindi G, Kloppel G, Couvelard P, Komminioth P, Korner M, Lopes JM, McNicol AM, Nilsson O, Perren A, Scarpa A, Scoazec JY, Wiedenmann B. TMN staging of mid and hindgut (neuro) endocrine tumors: a consensus proposal including a grading system. Virchows Archives. 2007: 451:757-762.

Sankhala K, Mita A, Kelly K, Mahalingam D, Giles F, Mita M. The emerging safety profile of mTOR inhibitors, a novel class of anticancer agents. Target Oncology 2009; 4 (2):135-42.

Sarbassov DD, Guertin DA, Ali SM, Sabatini DM. Phosphorylation and regulation of Akt/PKB by the rictor-mTOR complex. Science. 2005: Feb 18;307(5712):1098-101.

Shaw RJ. LKB1 and AMP-activated protein kinase control of mTOR signalling and growth. Acta Physiol 2009: May;196(1):65-80.

Simon R, Freidlin B, Rubinstein L, et al. Accelerated Titration Designs for Phase I Clinical. Trials in Oncology, Journal of the National Cancer Institute, (1997) Vol. 89, No. 15.

Svejda B, Kidd M, Kazberouk A, Lawrence B, Pfragner R, Modlin IM. Limitations in small intestinal neuroendocrine tumor therapy by mTor kinase inhibition reflect growth factor-mediated PI3K feedback loop activation via ERK1/2 and AKT. Cancer, 2011; epub ahead of print.

Turina, M, Christ-Crain, M, & Polk, HC, Jr. Diabetes and hyperglycemia: strict glycemic control. Critical Care Medicine, 2006: 34(9 Suppl): S291-S300.

Wald A. Sequential tests of statistical hypotheses. Annals of Mathematical Statistics. 1945; 16 (2):117-186.

Wen P, Macdonald, DR, Reardon, DA, et al. Updated response assessment criteria for high-grade gliomas: Response assessment in neuro-oncology working group. J Clin Oncol 2010; 28: 1963-1972.

EDMS Doc. Number: 20012337 - 16445273
