# Supplementary material for: A phase 2 study of an oral mTORC1/mTORC2 kinase inhibitor (CC-223) for non-pancreatic neuroendocrine tumors with or without carcinoid symptoms
Source: PLoS One. 2019 Sep 17;14(9):e0221994. doi: 10.1371/journal.pone.0221994 (PMC6748410; doi:10.1371/journal.pone.0221994)
Supplement: S3 File — (DOCX) [file pone.0221994.s003.docx]

**A phase 1/2 study of an oral mTORC1/mTORC2 kinase inhibitor (CC-223) for non-pancreatic neuroendocrine tumors with or without carcinoid symptoms**

Edward Wolin,^1*^ Alain Mita,^1^ Amit Mahipal,^2^ Tim Meyer,^3^ Johanna Bendell,^4^ John Nemunaitis,^5**^ Pam N Munster,^6^ Luis Paz-Ares,^7^ Ellen H Filvaroff,^8^ Shaoyi Li,^9^ Kristen Hege,^8^ Hans de Haan,^8^ and Monica Mita^1^

^1^Experimental Therapeutics Program, Samuel Oschin Comprehensive Cancer Institute, Cedars-Sinai Medical Center, Los Angeles, CA, USA; ^2^Phase 1 Clinical Trials Program, Mayo Clinic, Rochester, MN, USA; ^3^Experimental Cancer Medicine, University College Hospital, London, UK; ^4^GI Oncology Research, Sarah Cannon Research Institute/Tennessee Oncology, Nashville, TN, USA; ^5^Department of Oncology, MaryCrowley Cancer Research Center, Dallas, TX, USA; ^6^Early Phase Clinical Research Program, UCSF Helen Diller Family Comprehensive Cancer Center, San Francisco, CA, USA; ^7^Medical Oncology Department, Hospital Universitario 12 de Octubre, CNIO, Universidad Complutense and CiberOnc, Madrid, Spain; ^8^Translational Medicine, Celgene Corporation, San Francisco, CA, USA; ^9^Statistics, Celgene Corporation, Summit, NJ, USA

*Affiliation at the time the study was conducted. Currently at Tisch Cancer Institute, Mount Sinai School of Medicine, New York, NY, USA

**Affiliation at the time the study was conducted. Currently at University of Toledo, College of Medicine and Life Sciences, Ruppert Health Center, Toledo, OH, USA

# Supporting Information

## Checklist S1. CONSORT checklist.

## Protocol S1. Trial protocol.

## Figure S1. CONSORT diagram.

##
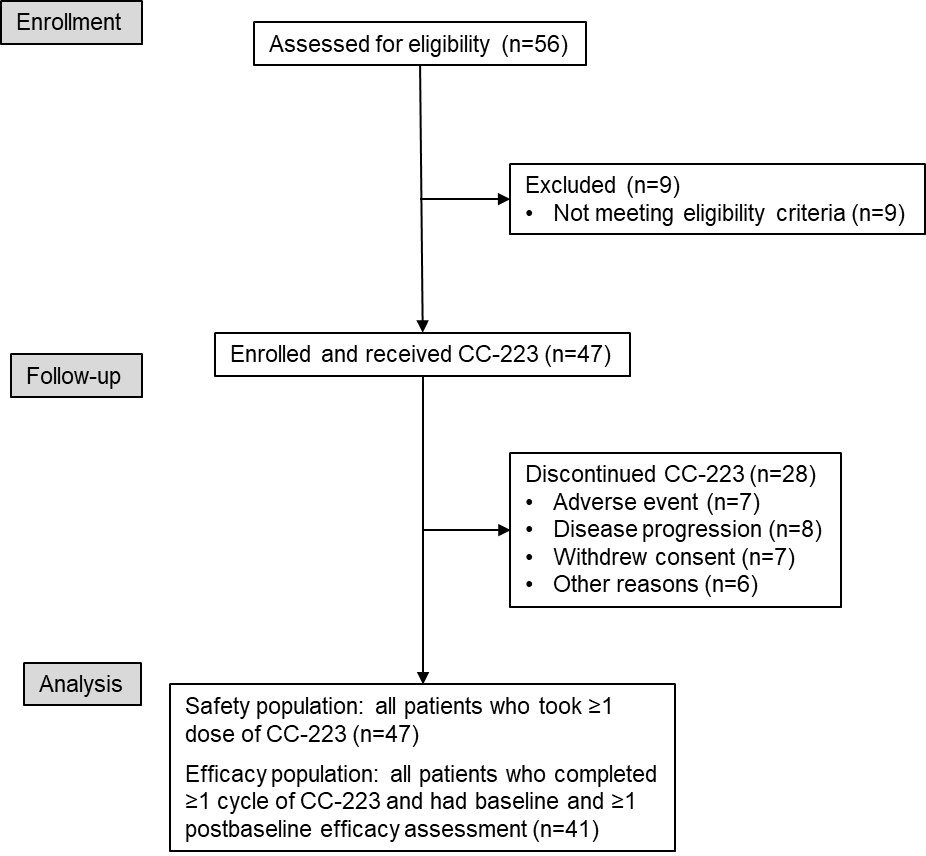


## Figure S2. CC-223 dose adjustments and duration, best overall RECIST version 1.1 and target lesion response, and carcinoid symptom improvement, for patients started at 45 mg/day (A) and 30 mg/day (B) CC-223.

Cycle, total treatment cycles completed; RECIST, best overall response; TL change (%), best target lesion change from baseline.

★, carcinoid symptomatic improvement; NE, not evaluable; PD, progressive disease; PR, partial response; RECIST, Response Evaluation Criteria In Solid Tumors; SD, stable disease.
