# Supplementary figures and images for: A phase 2 study of an oral mTORC1/mTORC2 kinase inhibitor (CC-223) for non-pancreatic neuroendocrine tumors with or without carcinoid symptoms
Source: PLoS One. 2019 Sep 17;14(9):e0221994. doi: 10.1371/journal.pone.0221994 (PMC6748410; doi:10.1371/journal.pone.0221994)

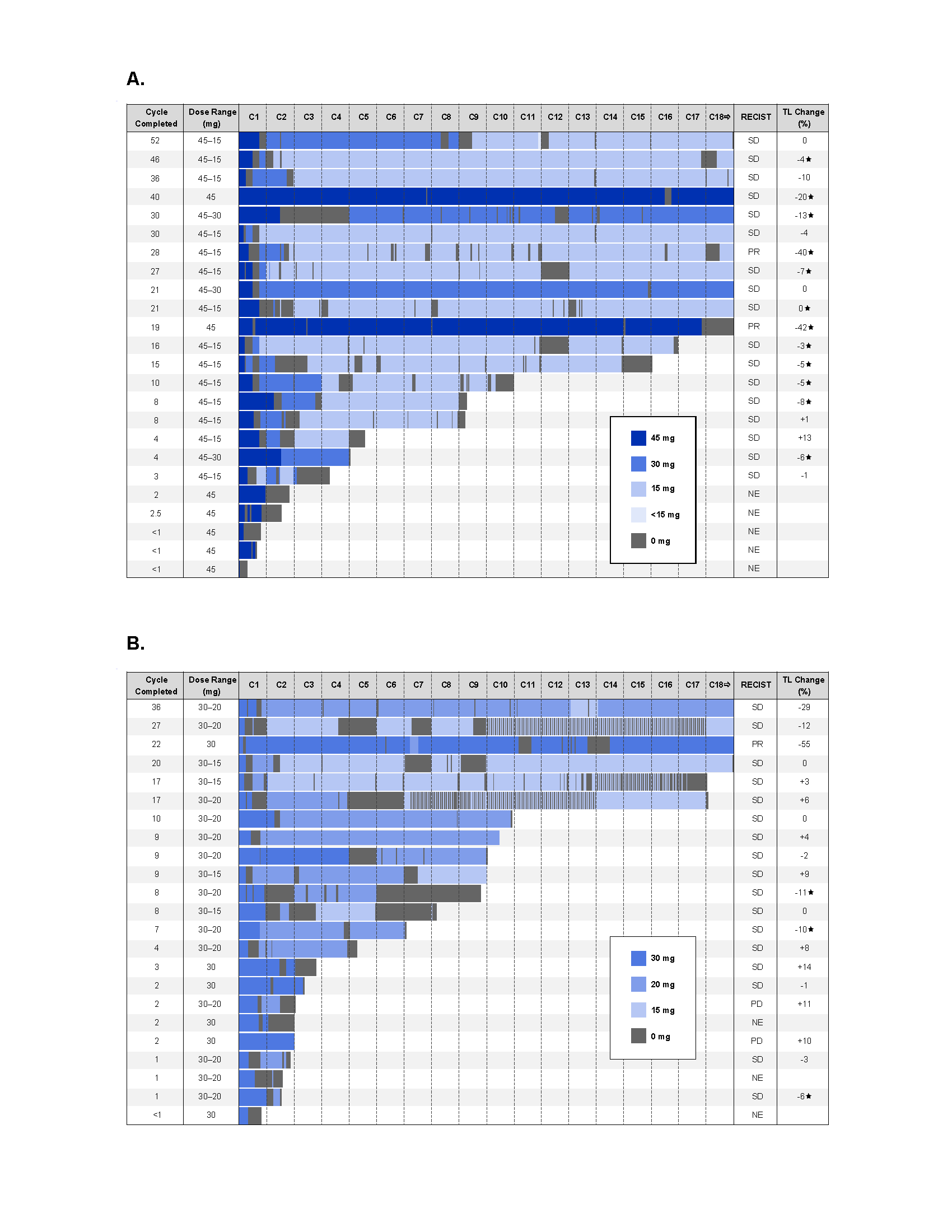

Supplement: S1 Fig — CC-223 dose adjustments and duration, best overall RECIST version 1.1 and target lesion response, and carcinoid symptom improvement, for patients started at 45 mg/day (A) and 30 mg/day (B) CC-223. Cycle, total treatment cycles completed; RECIST, best overall response; TL change (%), best target lesion change from baseline. ★, carcinoid symptomatic improvement; NE, not evaluable; PD, progressive disease; PR, partial response; RECIST, Response Evaluation Criteria In Solid Tumors; SD, stable disease. (TIF) [file pone.0221994.s004.tif]
